# Trends in mortality in people with heart failure and atrial fibrillation: a population-based cohort study

**DOI:** 10.1016/j.lanhl.2025.100734

**Published:** 2025-08-13

**Authors:** Nicholas R Jones, Margaret Smith, Yaling Yang, F D Richard Hobbs, Clare J Taylor

**Affiliations:** Nuffield Department of Primary Care Health Sciences, University of Oxford, Oxford, UK; Nuffield Department of Primary Care Health Sciences, University of Oxford, Oxford, UK; NIHR Oxford Biomedical Research Centre, Oxford, UK; Nuffield Department of Primary Care Health Sciences, University of Oxford, Oxford, UK; Nuffield Department of Primary Care Health Sciences, University of Oxford, Oxford, UK; Department of Applied Health Sciences, School of Health Sciences, College of Medicine and Health, University of Birmingham, Birmingham, UK

## Abstract

**Background:**

Atrial fibrillation and heart failure frequently coexist but the relative effect of atrial fibrillation on survival in people with heart failure, and vice versa, remains uncertain. We aimed to report contemporary estimates of mortality among people with atrial fibrillation and heart failure and analyse trends in mortality over time.

**Methods:**

We did a retrospective cohort study of adults aged 45 years or older in England, using primary care data from the Clinical Practice Research Datalink GOLD dataset and linked secondary care data (Hospital Episode Statistics and Office for National Statistics datasets), for a total follow-up period from Jan 1, 2000, to Dec 31, 2018. We recorded incident cases of heart failure and atrial fibrillation in primary or secondary care during the study period, as well as pre-existing cases at the study index date. Individuals were categorised as having both heart failure and atrial fibrillation, atrial fibrillation only, heart failure only, or neither condition, with heart failure and atrial fibrillation included in analyses as time-varying covariates. The primary outcome was all-cause mortality, as recorded in primary or secondary care. We report the incidence and hazard ratios for all-cause mortality by diagnosis status, median overall survival following diagnosis, and the cumulative probability of all-cause mortality from 3 months to 10 years of follow-up and by year of diagnosis to assess trends over time. Estimates of median survival and the cumulative probability of overall mortality were restricted to incident diagnoses during the study period, and calculated overall as well as by sex, age, and Index of Multiple Deprivation quintile.

**Findings:**

The cohort consisted of 2 381 941 people, including 100 132 initially diagnosed with heart failure only and 155 061 initially diagnosed with atrial fibrillation only by the study index date or during follow-up. By the end of follow-up, 74 470 people had been diagnosed with both conditions. 314 042 people died during follow-up, including 42 427 (57·0%) of those diagnosed with both heart failure and atrial fibrillation. In people diagnosed with both conditions during the study period (n=43 714), median overall survival was 3·15 years (95% CI 3·08–3·21), and the cumulative probability of mortality was 31·8% (95% CI 30·2–33·6) at 1 year, 61·4% (59·4–63·3) at 5 years, and 80·2% (78·3–82·1) at 10 years after both conditions had been diagnosed, representing significantly worse rates than for an initial diagnosis of either condition alone. Similarly, the risk-adjusted hazard of all-cause mortality was highest among people with both heart failure and atrial fibrillation. For the overall population, cumulative mortality probability estimates were unchanged over successive years of diagnosis for people with both heart failure and atrial fibrillation, while showing small improvements for people initially diagnosed with heart failure only (median reduction in 10-year cumulative probability of 3·8% [95% CI 1·4–6·1] between diagnosis years 2000 and 2008) or atrial fibrillation only (median reduction in 1-year cumulative mortality probability of 2·4% [0·5–4·2] between diagnosis years 2000 and 2017) and improvement over the long-term for people diagnosed with both conditions before age 65 years (median reduction in 10-year cumulative mortality probability of 14·5% [95% CI 3·8–25·2] between diagnosis years 2000 and 2008). For people with both conditions, median overall survival was significantly longer in the least deprived quintile (3·46 years [95% CI 3·31–3·59]; n=9275) than in the most deprived quintile (2·67 years [2·51–2·81]; n=6302). Median overall survival in each exposure group was similar between sexes after stratifying for age.

**Interpretation:**

Comorbid heart failure and atrial fibrillation was common and prognosis was poor, with no improvement in mortality estimates for diagnoses over time, and the worst survival in socially deprived groups.

**Funding:**

Wellcome Trust and the National Institute for Health and Care Research Collaboration for Leadership in Applied Health Research and Care Oxford.

## Introduction

There is a developing global twin epidemic of atrial fibrillation and heart failure,^1–3^ driven by ageing populations and the increasing prevalence of shared risk factors, including hypertension and obesity.^4,5^ Furthermore, the presence of either atrial fibrillation or heart failure increases the risk of developing the other condition.^6^ An analysis of individuals in the Framingham Heart Study found that more than half of people with newly diagnosed heart failure had pre-existing atrial fibrillation, and more than a third of those with newly diagnosed atrial fibrillation had pre-existing heart failure.^7^

Both atrial fibrillation and heart failure have been independently linked to an increased risk of all-cause mortality.^8,9^ Heart failure is associated with a particularly poor prognosis, with a median 5-year overall survival of around 50% among community cohorts.^10^ Pre-existing heart failure in people who subsequently develop atrial fibrillation is associated with an increased risk of mortality, and vice versa, although the reported effect sizes vary markedly between studies depending on the population characteristics.^6,11–14^

Previous studies of mortality in people with atrial fibrillation and heart failure have typically used secondary care datasets,^12,15,16^ or clinical trial datasets^13,14^ that might not be directly generalisable to wider community populations. Meanwhile, previous community-based studies might not be representative of current populations with atrial fibrillation and heart failure, given changing demographics over time and relatively small sample size.^7^ Furthermore, there are now several treatments available that have been shown to confer a prognostic benefit in heart failure.^17^ More recent community cohort studies have reported on changes in heart failure-related mortality among people with atrial fibrillation and in atrial fibrillation-related mortality among people with heart failure during the past two decades, but without reporting on changes in the prevalence of atrial fibrillation or heart failure over that time.^18,19^

In this study, we aimed to report the incidence and relative risk of all-cause mortality among people with both atrial fibrillation and heart failure, as well as for either condition alone, among a large English primary care cohort, to provide contemporary estimates of survival. We also report trends in mortality over time and in relation to age, sex, and social deprivation to examine how prognosis has changed with time.

## Methods

### Study design and participants

We did a retrospective cohort study using primary care data from general practices in England from the Clinical Practice Research Datalink (CPRD) GOLD dataset, and corresponding linked secondary care data from the Hospital Episode Statistics (hospitalisation data) and Office for National Statistics (mortality data based on civil registration) datasets.^20^ An Independent Scientific Advisory Committee (ISAC) reviewed the study protocol and approved the access to the CPRD (ISAC protocol number 19_125).

CPRD GOLD is a database of routinely collected data contributed by participating general practices using Vision software (In Practice Systems, Cegedim Healthcare Solutions). Extracted data include demographic information, coded diagnoses, prescriptions, and referrals. In July, 2013, 11·3 million patient records were held within CPRD GOLD from 674 general practices, and the data have been shown to be broadly representative of the wider UK population.^20^ We used CPRD GOLD to identify a large population-based cohort, including people diagnosed in primary or secondary care with atrial fibrillation, heart failure, or both conditions during the study period, as well as people with a pre-existing diagnosis of either condition or both conditions at the study index date.

People were included in the cohort if they were aged 45 years or older, eligible for data linkage (ie, from CPRD GOLD practices in England that have consented to participate in the CPRD linkage scheme), and registered for at least 1 year at an up-to-standard CPRD GOLD practice (per CPRD data quality criteria)^20^ between Jan 1, 2000, and Dec 31, 2018. Individuals were also required to have date of birth and sex codes, as well as patient and practice identification numbers. Individuals entered the cohort on the latest of the following dates, defined as their cohort entry date: Jan 1, 2000; the date of the person’s 45th birthday; the individual’s registration date plus 1 year; or the practice up-to-standard date plus 1 year. Individuals left the cohort on the earliest of the following dates: date of death; Dec 31, 2018; the date that practice data collection for the CPRD ended; the last date that linked data were available; or their transfer out of a CPRD registered practice. Thus the cohort follow-up period covered Jan 1, 2000 through to Dec 31, 2018.

### Procedures

We defined the date of diagnosis with atrial fibrillation or heart failure as the first date a corresponding diagnostic code had been entered into an individual’s primary care or secondary care record. We defined the date of diagnosis with heart failure and atrial fibrillation as the first date both conditions had been recorded if heart failure and atrial fibrillation were first diagnosed on the same date, or the first date that patients with prevalent atrial fibrillation or heart failure were diagnosed with the other condition (diagnostic codes are listed in the appendix [pp 10–12]). We included people with pre-existing atrial fibrillation or heart failure at the start of the study period and all incident cases during the study period. Individuals were categorised as having both heart failure and atrial fibrillation, atrial fibrillation without heart failure (atrial fibrillation only), heart failure without atrial fibrillation (heart failure only), or neither condition.

The primary outcome was all-cause mortality, as recorded in the CPRD, Hospital Episode Statistics, or Office for National Statistics records.

### Statistical analysis

We used descriptive statistics to compare the study population at the cohort entry (index) date, categorised by pre-existing atrial fibrillation and heart failure. Heart failure and atrial fibrillation were included in analyses as time-varying covariates, meaning that individuals could move between exposure groups across follow-up. In the main analyses, the atrial fibrillation only and heart failure only groups could include individuals who were initially diagnosed with either atrial fibrillation or heart failure alone but who were subsequently diagnosed with the other condition. Individuals with prevalent atrial fibrillation or heart failure who were diagnosed with the other condition were censored from their original group at the first date of having both conditions. The incidence of all-cause mortality was reported as the total number of deaths during follow-up by category of diagnosis. Median overall survival was calculated overall and by age, sex, and Index of Multiple Deprivation (IMD) quintile^21^ from the diagnosis date with heart failure or atrial fibrillation (or both conditions), excluding people diagnosed with atrial fibrillation or heart failure before the study index date. IMD quintile was derived from the CPRD through small-area level data linkage. The survival estimates and corresponding 95% CIs were calculated with the stci package in Stata (version 14). The likelihood ratio χ^2^ test was used to test for interactions between age and sex with median survival times.

We also calculated the cumulative probability of all-cause mortality at 3 months and 1, 2, 5, and 10 years of follow-up and by year of diagnosis, overall and by sex, age, and IMD quintile using the Kaplan–Meier product-limit estimate of the failure function, again excluding people diagnosed with atrial fibrillation or heart failure before the study index date. We present Kaplan–Meier curves as a descriptive summary of cumulative mortality probability in the different exposure groups. The curves were adjusted for sex and for a mean age of 76 years, given that this was the mean age of diagnosis with atrial fibrillation in the cohort during follow-up. Mean ages at diagnosis of atrial fibrillation (76 years) and heart failure (77 years) were similar in the cohort, but we chose the younger age to avoid over-adjusting for age. We also calculated the median difference in the cumulative probability of mortality between the earliest and most recent years of diagnosis by exposure groups, and calculated the 95% CI using the normal distribution. This was done for the overall cohort and by sex and age. Linear trends in the cumulative probability of mortality were investigated by fitting a weighted linear regression model, regressing the probability of mortality at 3 months and 1, 2, 5, and 10 years of follow-up against the year of diagnosis, with the weights inversely proportional to the variance in the mortality probability.

A Cox proportional hazards model was used to estimate the relative hazard of all-cause mortality during the study period by diagnosis status, first unadjusted, then adjusted for age and sex, then adjusted for age, sex, hypertension, type 1 or type 2 diabetes, history of stroke, thromboembolism, or vascular disease (including ischaemic heart disease, myocardial infarction, aortic plaque, peripheral arterial disease, or peripheral vascular disease), smoking status, and ethnicity. All covariates were identified from primary care electronic health records, with diagnoses and characteristics based on the most recent codes before the cohort index date where applicable. There was no limitation on how far before the index date the clinical codes could be entered to be considered relevant, although CPRD only began collecting data in 1987. Codes entered before the cohort index date were included to capture historical events such as a previous myocardial infarction or thromboembolism that might not have recurred during the study period but were risk factors for future cardiovascular events and death. Apart from sex and ethnicity, all covariates were considered as time-varying covariates and were updated at the time of a new diagnosis of either heart failure or atrial fibrillation. This included changes in the covariates between the index date and the heart failure and/or atrial fibrillation diagnosis date, or in the 90 days after a diagnosis. We also used a Cox model to report on the relative hazard of all-cause mortality based on the order in which heart failure and atrial fibrillation were diagnosed, including a subgroup analysis by sex and age categories.

The cohort included people with pre-existing heart failure or atrial fibrillation before cohort entry and these people were included in the mortality hazard modelling, but we excluded people with a diagnosis of heart failure or atrial fibrillation before the cohort index date when reporting on median survival and cumulative probability of mortality to avoid the risk of immortal time bias.

In a sensitivity analysis of the cumulative mortality probability modelling, we excluded people with heart failure or atrial fibrillation who went on to develop the other condition (atrial fibrillation or heart failure, respectively) by limiting to people who had either heart failure or atrial fibrillation only during the study period. As for the main analysis, we determined cumulative probability of all-cause mortality by year of diagnosis at 3 months and 1, 2, 5, and 10 years of follow-up for the overall groups, and assessed linear trends. As a secondary analysis, we report on observed short-term (3-month) all-cause mortality in relation to which health-care setting heart failure or atrial fibrillation was first diagnosed. In this analysis, first diagnosis was categorised as being in either the primary care record, secondary care record, or both records on the same date, depending on which database had the earliest recorded date of diagnosis. This analysis excluded people with a pre-existing diagnosis of either heart failure or atrial fibrillation at the study index date.

There were notable amounts of missing data for smoking status and ethnicity, which we felt were unlikely to be missing completely at random. We created a missing variable category for these data in the main analysis. Diagnostic codes were assumed to be complete. Having date of birth and sex codes was a prerequisite for inclusion in the study.

Statistical significance was interpreted on the basis of 95% CIs. The analyses were completed with Stata (version 14).

### Role of the funding source

The funders of the study had no role in study design, data collection, data analysis, data interpretation, or writing of the report.

## Results

The cohort consisted of 2 381 941 people, including 100 132 people initially diagnosed with heart failure only and 155 061 initially diagnosed with atrial fibrillation only, either before or during the study period. Of those initially diagnosed with heart failure only, 23 219 (23·2%) were subsequently diagnosed with atrial fibrillation; and of those initially diagnosed with atrial fibrillation only, 36 150 (23·3%) were subsequently diagnosed with heart failure. 15 101 people were diagnosed with heart failure and atrial fibrillation on the same date, 11 279 within the study period. By the end of the study period, 74 470 people had been diagnosed with heart failure and atrial fibrillation; 2 111 647 people were diagnosed with neither condition. Among people with a pre-existing condition at the cohort index date (n=87 874), 16 213 had heart failure and atrial fibrillation, 40 582 had atrial fibrillation only, and 31 079 had heart failure only. A complete flowchart of participant diagnoses over follow-up is provided in the appendix (p 5). Among people first diagnosed with heart failure and subsequently atrial fibrillation (n=23 219), the median time between the two diagnoses was 2·84 years (IQR 0·66–6·93). Similarly, among people first diagnosed with atrial fibrillation and then heart failure (n=36 150), the median time between diagnoses was 2·98 years (0·65–7·27).

151 383 people had heart failure and 193 381 had atrial fibrillation, with or without the other condition, before or during the study period. Of the 151 383 individuals with heart failure, 77 492 (51·2%) were first diagnosed in secondary care, compared with 67 032 (44·3%) in primary care. For the 193 381 individuals with atrial fibrillation, 94 654 (48·9%) were first diagnosed in secondary care, compared with 84 623 (43·8%) in primary care. The earliest diagnostic date was the same date in both the primary and secondary care records for 6859 (4·5%) people with heart failure and 14 104 (7·3%) people with atrial fibrillation.

The mean age at which both heart failure and atrial fibrillation had been diagnosed was 79·2 years (SD 9·9), compared with 75·9 years (11·0) for first diagnosis of atrial fibrillation and 77·0 years (11·1) for first diagnosis of heart failure. On average, female individuals were around 5 years older than male individuals at the time both conditions had been diagnosed or when either condition alone was diagnosed (appendix p 1).

Table 1 summarises baseline characteristics across groups categorised by pre-existing atrial fibrillation or heart failure at the cohort index date. Compared to people with atrial fibrillation only at the index date, those with heart failure with or without atrial fibrillation had greater burdens with regard to comorbidities, moderate to severe frailty, and prescribed medication. Among people with heart failure and atrial fibrillation at the index date, 5736 (35·4%) of 16 213 had six or more comorbidities, compared with 7065 (22·7%) of the 31 079 people with heart failure only, and 5252 (12·9%) of the 40 582 people with atrial fibrillation only. 7527 (46·4%) of the people with heart failure and atrial fibrillation at the index date were prescribed ten or more medications, compared with 11 988 (38·6%) in those with heart failure only, and 9853 (24·3%) in those with atrial fibrillation only. Among people diagnosed with atrial fibrillation either before the study index date or during the study period with a CHA_2_DS_2_-VASc score of at least 2 (indicating a moderate to high risk of stroke, for which oral anticoagulation therapy is generally recommended), a low proportion were prescribed an oral anticoagulant, particularly in the early years of follow-up, increasing to 8784 (55·3%) of 15 880 people with atrial fibrillation (with or without heart failure) by the end of follow-up (appendix p 1). In total, 121 399 (75·7%) of 160 305 people with atrial fibrillation diagnosed before or during the study period with a CHA_2_DS_2_-VASc score of at least 2 were prescribed an oral anticoagulant or antiplatelet (77 275 [48·2%] prescribed an antiplatelet, of whom 69 894 [90·4%] received aspirin).

In total, 314 042 deaths occurred during follow-up, including 42 427 deaths (57·0%) among the 74 470 people diagnosed with both heart failure and atrial fibrillation. Of the people with atrial fibrillation with or without heart failure, 81 983 (42·4%) of 193 381 died during follow-up, and of the people with heart failure with or without atrial fibrillation, 82 806 (54·7%) of 151 383 died during followup. Among people with neither condition at cohort entry, 191 680 (8·4%) of 2 294 067 died during follow-up. The median overall survival time for people diagnosed with heart failure and atrial fibrillation, restricting to those who received both diagnoses during the study period (n=43 714), was 3·15 years (95% CI 3·08–3·21), which was significantly shorter than for people initially diagnosed with heart failure only during the study period (n=63 912; 4·08 years [4·01–4·14]) and for people initially diagnosed with atrial fibrillation only during the study period (n=107 233; 6·55 years [6·46–6·65]; appendix p 3).

**Figure 1:**
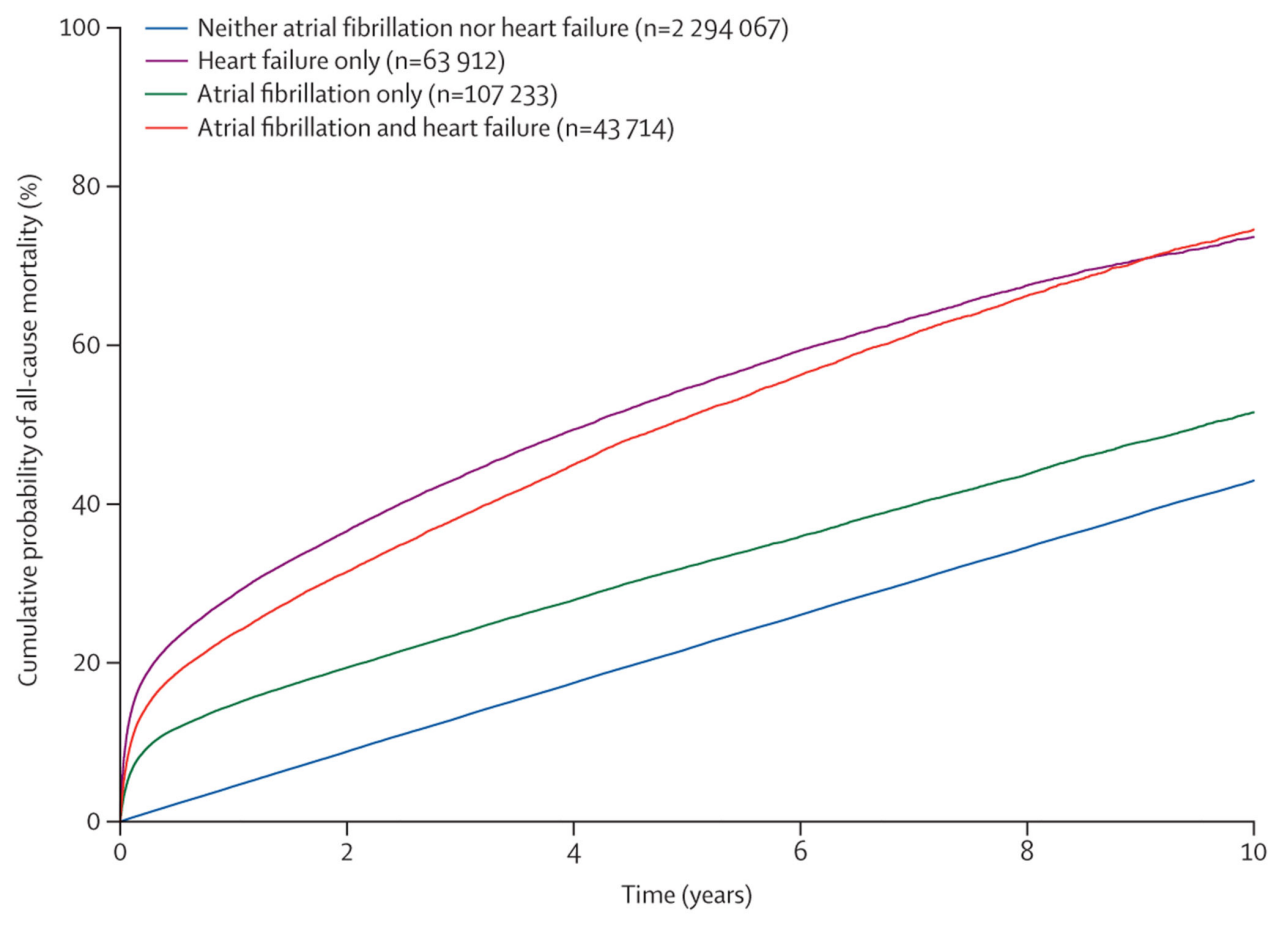
Curves showing the age-adjusted and sex-adjusted cumulative probability of all-cause mortality over time in people with heart failure and/or atrial fibrillation compared with people without either condition Heart failure and atrial fibrillation were included as time-varying covariates to allow individuals to move between exposure groups across follow-up, with analysis restricted to incident diagnoses during the study period (appendix p 5). People initially diagnosed with heart failure or atrial fibrillation alone but who were subsequently diagnosed with the other condition were included in the heart failure only and atrial fibrillation only groups, respectively; these individuals were censored from their original group at the time of the second diagnosis. The curves were adjusted for sex and for a mean age of 76 years, as the mean age of diagnosis with atrial fibrillation in the cohort during follow-up (appendix p 1). Curves were limited to a 10-year follow-up, consistent with the cumulative probability reporting and because there were limited survival data beyond this for people with heart failure and atrial fibrillation.

At 10 years of follow-up, the age-adjusted and sex-adjusted cumulative probability of all-cause mortality was highest among people diagnosed with heart failure and atrial fibrillation, closely followed by people initially diagnosed with heart failure only (figure 1). Similarly, in the unadjusted Cox model, the hazard of all-cause mortality over the total study period was highest among people with heart failure and atrial fibrillation (table 2). This was consistent in the age-adjusted and sex-adjusted model, and after adjusting for other cardiovascular risk factors, with the highest hazard in people with both conditions in the final adjusted model (hazard ratio [HR] 3·60 [95% CI 3·56–3·65]), followed by those initially diagnosed with heart failure only (2·98 [2·94–3·02]) and those initially diagnosed with atrial fibrillation only (1·77 [1·75–1·79]), representing significant differences in the relative hazard of all-cause mortality (table 2). Among people diagnosed with heart failure and atrial fibrillation, those diagnosed with both conditions on the same date had the highest hazard of death (HR 4·00 [3·92–4·10]) and those first diagnosed with atrial fibrillation who later developed heart failure had the lowest hazard of death (HR 3·36 [3·31–3·42]), representing a significant difference (appendix p 2).

From 1 year of follow-up, the cumulative probability of death was significantly higher for people with heart failure and atrial fibrillation at each follow-up time, compared to people initially diagnosed with either heart failure or atrial fibrillation only (table 3). The cumulative probability of death for people with heart failure and atrial fibrillation—measured from the date when both conditions had been diagnosed (the later date if recorded separately; n=43714)—was 20·2% (95% CI 18·8–21·8) at 3 months, 31·8% (30·2–33·6) at 1 year, 41·3% (39·5–43·2) at 2 years, 61·4% (59·4–63·3) at 5 years, and 80·2% (78·3–82·1) at 10 years. The corresponding estimates were 18·5% (17·5–19·6), 28·5% (27·2–29·7), 36·8% (35·5–38·2), 55·3% (53·8–56·7), and 74·1% (64·8–75·6), respectively, in people initially diagnosed with heart failure only (n=63 912), and 12·9% (12·1–13·7), 20·4% (19·4–21·4), 26·8% (25·8–27·9), 42·2% (40·9–43·4), and 60·8% (59·3–62·2), respectively, in people initially diagnosed with atrial fibrillation only (n=107 233).

Overall, mortality probability at each follow-up time was higher among female individuals than male individuals for each exposure group (table 3), although when comparing within subgroups of age, median overall survival times were similar between sexes (appendix p 3). Age at diagnosis was a key indicator of survival time, with a significant interaction between age subgroup and median overall survival (p<0·0001; appendix p 3). The cumulative probability of death significantly increased with age in all diagnosis groups, and was significantly higher in people with heart failure, with or without atrial fibrillation, compared to people initially diagnosed with atrial fibrillation only across age groups except in those aged 95 years and older, among whom mortality probability was similar between the exposure groups (table 3). Compared to people with neither heart failure nor atrial fibrillation, the relative hazard of all-cause mortality associated with heart failure and atrial fibrillation—diagnosed on the same or different dates—significantly decreased with age (appendix p 2). This reflected the increased all-cause mortality in those with neither condition among older age groups (appendix p 2).

**Figure 2:**
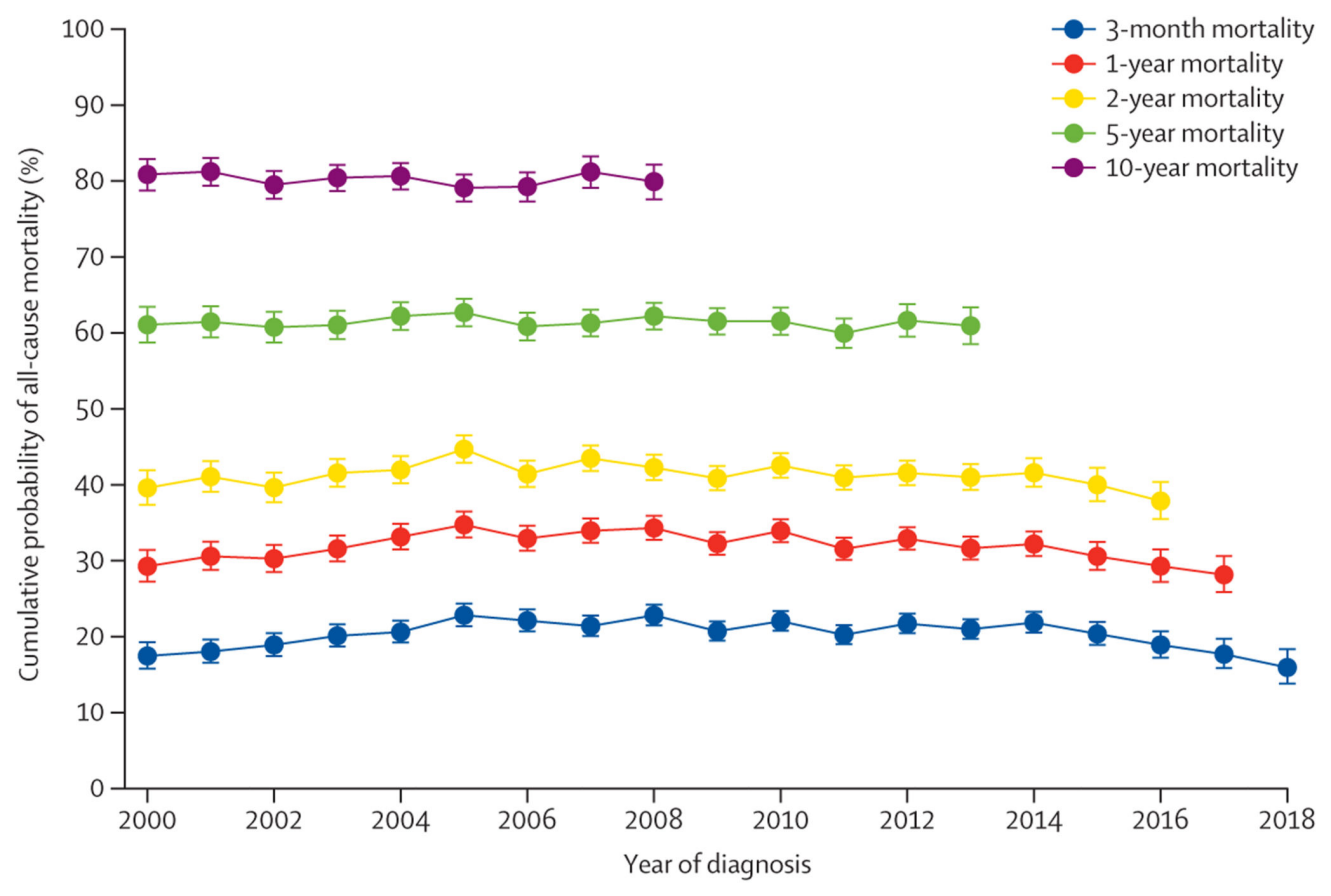
Trends in the cumulative probability of all-cause mortality at 3 months and 1, 2, 5, and 10 years of follow-up in people with both heart failure and atrial fibrillation (n=43 714) by year of diagnosis (2000–18) Bars represent 95% CIs. Year of diagnosis refers to the timepoint at which both conditions had been diagnosed (the later date if diagnosed on separate dates). Analysis was restricted to incident diagnoses during the study period (appendix p 5).

From earlier to later years of diagnosis, there was no improvement in estimations of short-term or long-term all-cause cumulative mortality probability for people with heart failure and atrial fibrillation (figure 2). For example, the cumulative probability of mortality at 1-year among people with heart failure and atrial fibrillation was 29·2% (95% CI 27·2 to 31·4) for those diagnosed with both conditions by the year 2000, and 28·2% (25·9 to 30·6) for those diagnosed with both conditions by 2017, with a median difference of –1·1% (95% CI –4·3 to 2·1; appendix p 4). The ages at which diagnoses of heart failure and atrial fibrillation were recorded remained constant for diagnoses at the start and end of the study period (appendix p 1).

When assessed by age, we observed a significant reduction in long-term mortality probability between the earliest and latest year of diagnosis among people diagnosed with both heart failure and atrial fibrillation before age 65 years (appendix p 4). In these individuals, the 5-year cumulative probability of mortality reduced by a median of 11·6% (95% CI 1·17–22·0), from 38·9% (95% CI 31·8–46·9) for those who had been diagnosed with both conditions by the year 2000 to 27·3% (20·9–35·2) for individuals diagnosed with both conditions by 2013, and the 10-year mortality probability reduced by a median of 14·5% (3·8–25·2), from 55·6% (47·8–63·7) for those diagnosed with both conditions by the year 2000 to 41·1% (34·3–48·6) for those diagnosed with both conditions by 2008. From the earliest to the latest year of diagnosis, there was a trend of improved short-term and long-term mortality probability in male individuals compared with female individuals with both heart failure and atrial fibrillation, although this only reached statistical significance for 5-year mortality, whereby there was a reduction in the 5-year cumulative probability of mortality among men diagnosed with both conditions by 2013 versus 2000 (median difference of –4·9% [95% CI –9·6 to –0·2]), compared with an increase in the 5-year cumulative probability of mortality among women diagnosed with both conditions by 2013 versus 2000 (difference of 5·2% [0·3 to 10·1]; appendix p 4).

For people initially diagnosed with heart failure only, those diagnosed in later years of the study period showed a small but statistically significant reduction in long-term mortality probability compared with those diagnosed in earlier years, with a median reduction in the 10-year cumulative probability of mortality of 3·8% (95% CI 1·4–6·1) for individuals diagnosed in 2008 versus those diagnosed in 2000 (appendix pp 4, 6). From the earliest to the latest year of diagnosis with heart failure, there was a small but statistically significant decrease in cumulative mortality probability after 1–10 years of follow-up among male individuals and people younger than 65 years, with no improvement in mortality probability for female individuals and people aged 75 years or older at diagnosis (appendix p 4). From the earliest to the latest year of diagnosis, there was a small but statistically significant improvement in 1-year cumulative mortality probability for people initially diagnosed with atrial fibrillation only (median reduction of 2·4% [0·5–4·2] between diagnosis years 2000 and 2017), but overall there was little improvement in mortality probability over time based on year of diagnosis, irrespective of age or sex (appendix pp 4, 7). In a sensitivity analysis of people with heart failure who did not later develop atrial fibrillation during the study period and vice versa, there were significant improvements in both short-term and long-term mortality estimates among both groups across successive years of diagnosis (appendix pp 8–9).

At the cohort index date, compared with individuals free of heart failure and atrial fibrillation, a higher proportion of people with heart failure, with or without atrial fibrillation, were in the most deprived IMD quintile and a lower proportion were in the least deprived quintile (table 1). Over follow-up, the median overall survival for people diagnosed with heart failure and atrial fibrillation was significantly longer by approximately 9 months in the least deprived quintile (3·46 years [95% CI 3·31–3·59], n=9275) than in the most deprived quintile (2·67 years [2·51–2·81], n=6302), which increased to a difference of more than 3 and a half years among those diagnosed with both conditions before the age of 75 years (least deprived quintile, 8·68 years [7·81–9·67], n=2356; most deprived, 5·07 years [4·50–5·48], n=2185). These directional differences in median overall survival were also observed in relation to IMD quintile among people who had heart failure or atrial fibrillation independently at diagnosis (appendix p 3).

**Figure 3:**
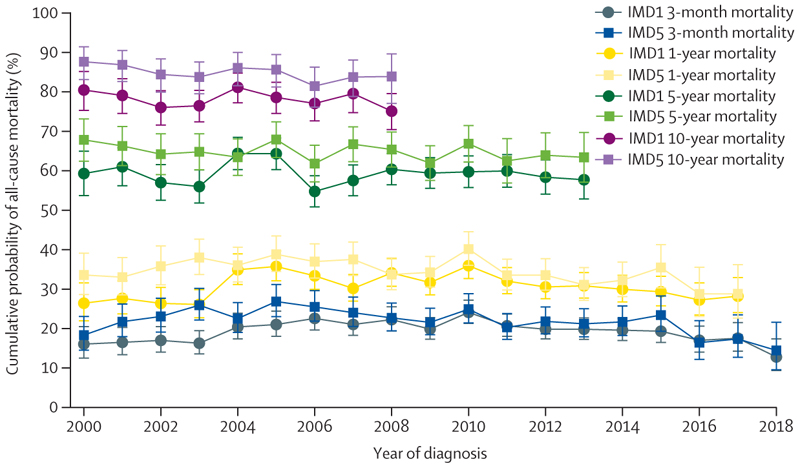
Comparison of trends in the short-term and long-term cumulative probability of all-cause mortality by year of diagnosis (2000–18) in people with both heart failure and atrial fibrillation (n=43 714), across areas of least and most deprivation Bars represent 95% CIs. Least deprived quintile (IMD 1) is represented by a circle and most deprived quintile (IMD 5) by a square. Year of diagnosis refers to the timepoint at which both conditions had been diagnosed (the later date if diagnosed on separate dates). Analysis was restricted to incident diagnoses during the study period (appendix p 5). IMD=Index of Multiple Deprivation.

From earlier to later years of diagnosis among people with both heart failure and atrial fibrillation, there was generally little change in short-term and long-term estimates of cumulative mortality probability for the least and most deprived groups. We observed a consistent trend of lower mortality probability among people in the least deprived IMD quintile compared to the most deprived quintile, although this difference was not statistically significant for most diagnosis years and follow-up timepoints (figure 3).

Excluding people with a pre-existing diagnosis of either heart failure or atrial fibrillation at the study index date, there were 16 734 (12·8%) deaths within 3 months of a diagnosis of atrial fibrillation (n=130 457; including people with or without heart failure), and 19 840 (20·7%) deaths within 3 months of a diagnosis of heart failure (n=95 840; including people with or without atrial fibrillation). Short-term mortality was higher among people whose diagnosis of atrial fibrillation or heart failure was first recorded in secondary care, than among those whose earliest diagnostic code was in primary care. Among 48 737 people with atrial fibrillation (with or without heart failure) first coded in primary care, 1214 (2·5%) died within 3 months of diagnosis, compared with 14 992 (21·2%) of 70 758 whose earliest code was in secondary care. 528 (4·8%) of 10 962 individuals had atrial fibrillation coded on the same date in both primary and secondary care. For people with heart failure (with or without atrial fibrillation), 3731 (10·9%) of 34 178 with the earliest diagnostic code in primary care died within 3 months of diagnosis, compared with 15 533 (27·4%) of 56 718 with the earliest diagnostic code in secondary care. 576 (11·7%) of 4944 people had heart failure coded on the same date in both primary and secondary care.

## Discussion

Overall, close to a third of people with both atrial fibrillation and heart failure were estimated to die within a year of having both diagnoses, and approximately 60% within the first 5 years, with no improvement in the probability of all-cause mortality in this group across successive years of diagnosis over nearly 20 years of follow-up. Age-stratified median overall survival was similar among male and female individuals. People with heart failure, with or without atrial fibrillation, were more likely to live in areas of greatest deprivation than those with neither condition, which in turn was associated with decreased overall survival. Additionally, in people with both conditions, the observable trend of higher mortality probability in the most deprived quintile versus the least deprived quintile did not change across follow-up.

Between the earliest and latest years of diagnosis, our results showed a significant improvement in long-term mortality probability for people diagnosed with both heart failure and atrial fibrillation before age 65 years, and a trend of improved mortality probability among male individuals with heart failure and atrial fibrillation, which wasn’t observed in female individuals. In the main analysis, people initially diagnosed with heart failure only, including those who subsequently developed atrial fibrillation, had modest reductions in the probability of mortality across successive years of diagnosis; whereas, in sensitivity analysis, there were significant improvements for the group with heart failure who did not develop subsequent atrial fibrillation during follow-up and vice versa, highlighting the negative effect on survival of comorbid heart failure and atrial fibrillation.

All-cause mortality is known to be high in people with heart failure, with overall survival previously estimated at 75·9% (95% CI 75·5–76·3) at 1 year and 45·5% (45·1–46·0) at 5 years after diagnosis based on primary care data.^9^ In the UK, the National Heart Failure Audit of 2018–19 data reported that for people admitted to hospital with heart failure, in-hospital mortality was 9·1%, with a further 14·9% dying within 30 days of discharge, and 1-year mortality was 32%.^22^ A recent UK population-wide analysis similarly reported 1-year overall survival of 32·2% following hospital admission with heart failure, and that this remained constant between 2019 and 2022.^23^ More than half (51·2%) of individuals with heart failure in our cohort were first diagnosed in hospital, which is likely to explain the similarly poor short-term mortality estimates observed in our cohort, which largely reflect death among people diagnosed in hospital. Our longitudinal analysis indicated limited improvement in these mortality estimates over time, while also highlighting the negative effect of comorbid atrial fibrillation.

Atrial fibrillation is associated with an increased risk of death, particularly from cardiovascular causes such as stroke, ischaemic heart disease, and myocardial infarction. This association is likely to reflect both the increased risk of embolic events in atrial fibrillation but also the association of atrial fibrillation with other cardiovascular risk factors, such as hypertension. A recent European registry study included 14 964 people with heart failure (mean age 66 years) and found overall atrial fibrillation to be associated with an increased risk of all-cause mortality and hospitalisation for heart failure over 2 years of follow-up. However, after multivariable adjustment, this association between atrial fibrillation and all-cause mortality and hospitalisation for heart failure was limited to people with heart failure with preserved ejection fraction or heart failure with mildly reduced ejection fraction and was not significant among people with heart failure with reduced ejection fraction.^12^ A systematic review published in 2009 reported that people with heart failure and comorbid atrial fibrillation were at increased risk of all-cause mortality compared to those with normal sinus rhythm, irrespective of left ventricular ejection fraction, but most studies only had short-term followup.^24^ By contrast, our study reports on long-term survival in people with heart failure and atrial fibrillation and in a community setting, reflecting where the majority of patients are treated. Additionally, in contrast to most clinical trials in people with heart failure,^25,26^ our population with heart failure and atrial fibrillation was older with a high burden of comorbid disease, and therefore more representative of people with heart failure in the community. Two recent studies reported on heart failure-related mortality among people with atrial fibrillation and atrial fibrillation-related mortality among people with heart failure, both within in the USA between 1999 and 2020.^18,19^ Age-adjusted annual atrial fibrillation-related mortality among people with heart failure and the absolute prevalence of heart failure-related deaths among people with atrial fibrillation both increased over time, but neither study accounted for changes in prevalence of the underlying conditions over time.

Although randomised trials have shown that prognostically beneficial treatments are available for people with heart failure,^27^ our study found that, at a population level, mortality estimates remained almost static in people initially diagnosed with heart failure only, although there were small improvements in long-term estimates, as well as among men and people diagnosed before age 75 years. Similar results have been reported among heart failure cohorts in the USA and the UK.^9,23,28^ By contrast, improvements in survival have been observed for people with heart failure in Denmark over a similar time period.^29^ The lack of improvement in survival might reflect the complexities of how, and for whom, evidence-based heart failure interventions are incorporated into practice.

Uniquely, our study shows the trends in all-cause mortality for people with heart failure and atrial fibrillation over an extended period. Our data come from a large database of primary care and linked secondary records in England, within a nationalised health service, and are therefore likely to be broadly representative of the national population and other high-income settings.

Categorisation of heart failure by impairment of left ventricular ejection fraction informs the current approach to heart failure treatment,^30^ but in our cohort there was an insufficient number of people with an ejection fraction coded to allow for an analysis on this basis. This is an important omission; atrial fibrillation has been specifically linked to heart failure with preserved ejection fraction,^7^ perhaps because increased filling pressures in heart failure with preserved ejection fraction result in dilatation of the left atrium or because left ventricular fibrosis occurs in people with atrial fibrillation because of the tendency for increased heart rates.^31^ Age-adjusted overall survival is typically worse in heart failure with reduced ejection fraction than in heart failure with preserved ejection fraction.^10^ We were also unable to report on whether atrial fibrillation was paroxysmal, persistent, or permanent due to incomplete coding.

Overall, our data relied on diagnostic coding, which might have led to misclassification in some situations. However, ICD-10 codes have been shown to have high positive predictive value for cardiovascular disease.^32^ Furthermore, we believe the use of a combination of primary and secondary care codes reduced the risk of misclassification, meaning it is unlikely misclassification would have altered our summary findings. We also did not report on the role of medication or other treatment modalities other than anticoagulation, despite the importance of these when interpreting trends in mortality. Overall, the proportion of people with atrial fibrillation with an indication for oral anticoagulation who were prescribed an oral anticoagulant was low, but aspirin was recommended as an alternative for stroke prevention in atrial fibrillation up until 2014, and 75·7% of those eligible were prescribed an oral anticoagulant or antiplatelet (mostly aspirin in those prescribed an antiplatelet) across the study period. Future research specifically designed to assess the uptake and effect of medication with proven prognostic benefits in heart failure and atrial fibrillation would be required to assess the influence of such medications on mortality.

Our study did not explore cause of death and some individuals will have died during an acute hospital admission in which heart failure or atrial fibrillation were diagnosed but were not the primary illness. There was also a risk of immortal time bias, as people who might have suspected heart failure detected in primary care would normally have the diagnosis confirmed in a secondary care clinic and so would need to live long enough to have that assessment, though these numbers would have likely been small and any resulting bias would likely lead to an underestimation of mortality risk within our cohort, further supporting the high mortality rates observed. Additionally, although we accounted for a range of key covariates, there is the possibility that other unmeasured factors, including treatments beyond anticoagulation, might have influenced mortality estimates. The lack of improvement in mortality probability over successive years of diagnosis in people with heart failure and atrial fibrillation might be at least partly due to residual confounding.

Improving prognosis among older individuals who typically have multiple long-term health conditions is challenging and resource intensive, requiring a holistic and generalist approach to optimal treatment. For some people, the aim of treatment will be improved quality of life rather than prolonged survival. Nonetheless, awareness of the prognostic importance of comorbid heart failure and atrial fibrillation in people with either condition alone might help to improve person-centred care, such as identifying suitable individuals with atrial fibrillation and heart failure for rhythm control therapy at an early stage. There are now a number of medications with proven prognostic benefit in heart failure, and optimising prescribing of these medications at scale in the community could also help to ensure all people can equally benefit. In particular, our data suggest health policy makers need to consider how prescribing of these medications can be done in a manner that aims to reduce health inequalities and close the survival gap that exists between the least and most deprived areas among people with heart failure and atrial fibrillation.

In summary, we found that comorbid heart failure and atrial fibrillation was common and associated with poor prognosis, with all-cause mortality estimates largely unchanged overall for diagnoses recorded across nearly 20 years of follow-up, and a persistent gap in survival based on deprivation status.

## Supplementary Material

Supplementary appendix

## Figures and Tables

**Table 1:** Baseline characteristics according to pre-existing atrial fibrillation and/or heart failure at the index date

	Neither atrial fibrillation nor heart failure	Heart failure only	Atrial fibrillation only	Atrial fibrillation and heart failure	Total
Number of individuals	2 294 067	31 079	40 582	16 213	2 381 941
Age, years	56·4 (12·2)	75·9 (12·2)	72·6 (12·9)	78·2 (10·9)	57·0 (12·7)
Sex					
Male	1109 577 (48·4%)	14 163 (45·6%)	21 585 (53·2%)	7675 (47·3%)	1153 000 (48·4%)
Female	1184490 (51·6%)	16 916 (54·4%)	18 997 (46·8%)	8538 (52·7%)	1 228 941 (51·6%)
Ethnicity					
White	1615 304 (70·4%)	25 687 (82·7%)	36 117 (89·0%)	14 289 (88·1%)	1691397 (71·0%)
Black	27 799 (1·2%)	382 (1·2%)	205 (0·5%)	92 (0·6%)	28 478 (1·2%)
Asian	45 131 (2·0%)	636 (2·0%)	395 (1·0%)	155 (1·0%)	46 317 (1·9%)
Mixed or Other	28 069 (1·2%)	339 (1·1%)	387 (1·0%)	128 (0·8%)	28 923 (1·2%)
Unknown	577 764 (25·2%)	4035 (13·0%)	3478 (8·6%)	1549 (9·6%)	586 826 (24·6%)
IMD quintile					
1 (least deprived)	584 985 (25·5%)	5901 (19·0%)	10 199 (25·2%)	3321 (20·5%)	604 406 (25·4%)
2	535 279 (23·3%)	6752 (21·7%)	9773 (24·1%)	3768 (23·3%)	555 572 (23·3%)
3	467 923 (20·4%)	6703 (21·6%)	8738 (21·5%)	3463 (21·4%)	486 827 (20·5%)
4	421 403 (18·4%)	6559 (21·1%)	7178 (17·7%)	3291 (20·3%)	438 431 (18·4%)
5 (most deprived)	282 481 (12·3%)	5131 (16·5%)	4653 (11·5%)	2352 (14·5%)	294 617 (12·4%)
Missing	1996 (<0·1%)	33 (0·1%)	41 (0·1%)	18 (0·1%)	2088 (<0·1%)
Smoking status					
Non-smoker	1063 899 (46·4%)	13 386 (43·1%)	19 305 (47·6%)	7348 (45·3%)	1103 938 (46·3%)
Current smoker	466 287 (20·3%)	4157 (13·4%)	4444 (11·0%)	1560 (9·6%)	476 448 (20·0%)
Ex-smoker	451 902 (19·7%)	8926 (28·7%)	12 038 (29·7%)	5022 (31·0%)	477 888 (20·1%)
Missing	311 979 (13·6%)	4610 (14·8%)	4795 (11·8%)	2283 (14·1%)	323 667 (13·6%)
Units of alcohol per week	7·8 (12·0)	4·8 (9·8)	7·0 (12·1)	5·4 (10·8)	7·7 (12·0)
BMI, kg/m^2^	26·6 (4·8)	27·7 (5·2)	26·8 (4·9)	26·9 (5·3)	26·6 (4·8)
Comorbidities*					
Chronic kidney disease	22 035 (1·0%)	1956 (6·3%)	2351 (5·8%)	1857 (11·5%)	28 199 (1·2%)
Type 1 or type 2 diabetes	112 425 (4·9%)	5585 (18·0%)	4578 (11·3%)	2949 (18·2%)	125 537 (5·3%)
Hypertension	427 629 (18·6%)	14 190 (45·7%)	18 272 (45·0%)	7871 (48·5%)	467 962 (19·6%)
Liver disease	9699 (0·4%)	200 (0·6%)	238 (0·6%)	81 (0·5%)	10 218 (0·4%)
Migraine	128 596 (5·6%)	1007 (3·2%)	1599 (3·9%)	466 (2·9%)	131 668 (5·5%)
Thromboembolism	13 806 (0·6%)	999 (3·2%)	1047 (2·6%)	729 (4·5%)	16 581 (0·7%)
Vascular disease†	59 571 (2·6%)	9177 (29·5%)	4962 (12·2%)	3882 (23·9%)	77 592 (3·3%)
Number of comorbidities					
0–1	1661 490 (72·4%)	2739 (8·8%)	7466 (18·4%)	720 (4·4%)	1 672 415 (70·2%)
2–5	601 799 (26·2%)	21 275 (68·5%)	27 864 (68·7%)	9757 (60·2%)	660 695 (27·7%)
6–9	30 250 (1·3%)	6708 (21·6%)	5040 (12·4%)	5280 (32·6%)	47278 (2·0%)
≥10	528 (<0·1%)	357 (1·1%)	212 (0·5%)	456 (2·8%)	1553 (0·1%)
Frailty category (electronic Frailty Index)					
Fit	2 131 093 (92·9%)	12 528 (40·3%)	23 030 (56·7%)	4417 (27·2%)	2 171 068 (91·1%)
Mild frailty	150 405 (6·6%)	14 406 (46·4%)	14 714 (36·3%)	8233 (50·8%)	187 758 (7·9%)
Moderate frailty	11 943 (0·5%)	3726 (12·0%)	2572 (6·3%)	3077 (19·0%)	21 318 (0·9%)
Severe frailty	626 (<0·1%)	419 (1·3%)	266 (0·7%)	486 (3·0%)	1797 (0·1%)
Number of different medications prescribed					
0–1	1 207 068 (52·6%)	1488 (4·8%)	4017 (9·9%)	395 (2·4%)	1 212 968 (50·9%)
2–5	737 001 (32·1%)	7043 (22·7%)	13 800 (34·0%)	2721 (16·8%)	760 565 (31·9%)
6–9	239 729 (10·4%)	10 560 (34·0%)	12 912 (31·8%)	5570 (34·4%)	268 771 (11·3%)
≥10	110 269 (4·8%)	11 988 (38·6%)	9853 (24·3%)	7527 (46·4%)	139 637 (5·9%)

Data are mean (SD) or n (%). IMD=index of multiple deprivation.

* All listed conditions were identified based on diagnostic codes in the patient’s electronic health record; additional cases of chronic kidney disease based on laboratory data (ie, estimated glomerular filtration rate or urine albumin-to-creatinine ratio) without a code for chronic kidney disease were not counted.

† Vascular disease included codes for ischaemic heart disease, myocardial infarction, aortic plaque, peripheral vascular disease, or peripheral arterial disease.

**Table 2:** Cox regression model showing the hazard of all-cause mortality during the study period by heart failure and atrial fibrillation status

	Number of participants	Number of deaths (% of participants)	Hazard ratio (95% CI)		
			Unadjusted model	Model 1	Model 2
Neither heart failure nor atrial fibrillation	2 294 067	191 680 (8·4%)	1 (ref)	1 (ref)	1 (ref)
Heart failure only	94 991	40 379 (42·5%)	12·00 (11·87–12·13)	3·07 (3·04–3·11)	2·98 (2·94–3·02)
Atrial fibrillation only	147 815	39 556 (26·8%)	6·14 (6·08–6·21)	1·75 (1·73–1·77)	1·77 (1·75–1·79)
Heart failure and atrial fibrillation	74 470	42 427 (57·0%)	17·94 (17·75–18·13)	3·68 (3·64–3·73)	3·60 (3·56–3·65)

Model 1 was adjusted for age and sex. Model 2 was adjusted for age, sex, hypertension, type 1 or type 2 diabetes, history of stroke, thromboembolism, or vascular disease (including ischaemic heart disease, myocardial infarction, aortic plaque, peripheral vascular disease, or peripheral arterial disease), smoking status, and ethnicity. Apart from sex and ethnicity, all covariates were considered as time-dependent covariates and were updated at the time of a new diagnosis of either heart failure or atrial fibrillation. Heart failure and atrial fibrillation were also included as time-varying covariates to allow individuals to move between exposure groups across follow-up. People who had already been diagnosed with heart failure or atrial fibrillation, or both, by the time of cohort entry were included in the analysis (appendix p 5). People initially diagnosed with heart failure or atrial fibrillation alone, either before or during follow-up, but who subsequently developed the other condition during the follow-up period were included in the heart failure only and atrial fibrillation only groups, respectively, as well as in the heart failure and atrial fibrillation group; these individuals were censored from their original group at the time of the second diagnosis.

**Table 3:** Cumulative probability of all-cause mortality at 3 months and 1, 2, 5, and 10 years of follow-up in people with heart failure and/or atrial fibrillation, stratified by sex and age

	3 months	1 year	2 years	5 years	10 years
Heart failure and atrial fibrillation* (n=43 714)					
Overall	20·2 (18·8–21·8)	31·8 (30·2–33·6)	41·3 (39·5–43·2)	61·4 (59·4–63·3)	80·2 (78·3–82·1)
Sex					
Female	22·1 (20·0–24·4)	34·0 (31·6–36·6)	43·5 (40·9–46·2)	63·8 (61·1–66·6)	81·9 (79·2–84·5)
Male	18·5 (16·5–20·6)	29·8 (27·4–32·2)	39·2 (36·7–41·8)	59·0 (56·3–61·8)	78·6 (75·9–81·3)
Age, years					
<65	8·8 (7·9–9·7)	13·8 (12·8–15·0)	18·5 (17·3–19·7)	28·9 (27·3–30·5)	43·7 (41·3–46·1)
65–74	13·6 (12·9–14·3)	21·2 (20·4–22·1)	27·8 (26·8–28·8)	43·1 (41·9–44·3)	65·3 (63·6–67·0)
75–84	19·5 (18·9–20·1)	30·7 (30·0–31·4)	39·5 (38·7–40·3)	61·3 (60·4–62·2)	84·4 (83·3–85·4)
85–94	30·5 (29·7–31·4)	45·7 (44·8–46·7)	58·1 (57·1–59·1)	81·8 (80·8–82·8)	96·5 (95·5–97·4)
≥95	44·1 (41·1–47·3)	63·1 (59·9–66·4)	77·7 (74·5–80·7)	96·2 (93·8–97·8)	NA
Atrial fibrillation only (n=107 233)					
Overall	12·9 (12·1–13·7)	20·4 (19·4–21·4)	26·8 (25·8–27·9)	42·2 (40·9–43·4)	60·8 (59·3–62·2)
Sex					
Female	14·3 (13·1–15·6)	22·1 (20·7–23·6)	28·8 (27·3–30·4)	44·8 (43·0–46·6)	63·7 (61·7–65·7)
Male	11·5 (10·4–12·6)	18·7 (17·5–20·1)	24·9 (23·5–26·4)	39·7 (38·0–41·4)	57·9 (56·0–59·9)
Age, years					
<65	4·7 (4·4–5·0)	7·6 (7·2–7·9)	9·8 (9·4–10·2)	14·7 (14·2–15·3)	22·4 (21·6–23·3)
65–74	7·9 (7·6–8·2)	13·0 (12·6–13·4)	17·0 (16·6–17·5)	27·6 (27·0–28·2)	44·2 (43·3–45·2)
75–84	13·2 (12·9–13·6)	21·4 (21·0–21·8)	28·4 (27·9–28·9)	46·4 (45·7–47·0)	71·3 (70·4–72·2)
85–94	22·9 (22·2–23·5)	36·1 (35·4–36·9)	47·3 (46·5–48·1)	72·2 (71·3–73·1)	93·3 (92·1–94·1)
≥95	37·7 (35·2–40·3)	57·8 (55·0–60·6)	71·7 (68·9–74·5)	93·1 (90·7–95·0)	NA
Heart failure only (n=63 912)					
Overall	18·5 (17·5–19·6)	28·5 (27·2–29·7)	36·8 (35·5–38·2)	55·3 (53·8–56·7)	74·1 (64·8–75·6)
Sex					
Female	19·9 (18·4–21·6)	30·3 (28·6–32·2)	38·8 (37·0–40·8)	57·8 (55·7–59·9)	76·8 (74·7–78·8)
Male	17·1 (15·7–18·7)	26·7 (25·0–28·4)	34·9 (33·0–36·8)	52·9 (50·9–55·0)	71·6 (69·5–73·7)
Age, years					
<65	9·5 (9·0–10·0)	13·4 (12·8–14·1)	17·3 (16·6–18·0)	27·0 (26·1–27·9)	41·0 (39·7–42·3)
65–74	13·5 (12·9–14·0)	20·7 (20·0–21·4)	26·9 (26·2–27·7)	41·5 (40·6–42·4)	61·5 (60·4–62·6)
75–84	18·6 (18·1–19·1)	28·9 (28·2–29·5)	37·6 (36·9–38·3)	58·2 (57·4–59·0)	80·9 (80·0–81·7)
85–94	26·7 (25·9–27·5)	41·5 (40·6–42·4)	53·5 (52·6–54·5)	77·5 (76·5–78·5)	94·9 (94·1–95·7)
≥95	40·3 (37·5–43·2)	59·5 (56·5–62·5)	74·4 (71·5–77·2)	94·5 (92·5–96·4)	NA

Each cell reports the probability of death in those at risk up to each respective follow-up time. Heart failure and atrial fibrillation were included as time-varying covariates, with analysis restricted to incident diagnoses during the study period (appendix p 5). People initially diagnosed with heart failure or atrial fibrillation alone but who were subsequently diagnosed with the other condition were included in the heart failure only and atrial fibrillation only groups, respectively; these individuals were censored from their original group at the time of the second diagnosis. NA=not available (insufficient data for analysis).

* Follow-up represents the time at which both conditions had been diagnosed, which was the later date for diagnoses on separate dates.

## Data Availability

The data used in this study are available to other researchers through the Clinical Practice Research Datalink (CPRD; https://www.cprd.com/data-access) but not publicly available. CPRD provides de-identified patient data from electronic health records of participating primary care surgeries in the UK. The data were made available to our team for this specific research project following review of our protocol by an Independent Scientific Advisory Committee (ISAC protocol number 19_125). No additional documents have been published alongside this manuscript.

## Research in context

### Evidence before this study

We searched PubMed and Google Scholar for large-scale, observational, and community-based studies reporting on mortality among people with heart failure and atrial fibrillation from Jan 1, 2000, to March 31, 2025, without language restrictions. Limiting to terms in the title or abstract, we combined “heart failure” AND “atrial fibrillation” along with terms for mortality (“surviv*”, “died”, “death”, “mortality”, “outcome*”, or “epidemiology”) and terms for observational studies (“retrospective”, “cohort”, “observational”, “regist*”, or “database”). We found one report on 55917 atrial fibrillation-related deaths among individuals who had comorbid heart failure in the USA between 1999 and 2020, based on data from the Centers for Disease Control and Prevention database (*J Investig Med* 2024; **72:** 13–16). In that study, age-adjusted atrial fibrillation-related mortality increased over the study period, particularly among male individuals. The age-adjusted atrial-fibrillation related mortality was higher among older adults (aged 65–84 years) than younger adults (aged 35–64 years), but the average annual percentage change in mortality was higher among younger adults across follow-up. A second retrospective cohort study, not identified in our search, reported on heart failure-related mortality among people with atrial fibrillation between 1999 and 2000 in the USA using data from the Centers for Disease Control and Prevention’s Wide-Ranging Online Data for Epidemiological Research (*J Am Heart Assoc* 2024; **13:** e033897). The study reported 916 685 heart failure-related deaths across the study period, with an increase in the absolute number of heart failure-related deaths across the study period and an increase in age-adjusted mortality from 8·15 deaths per 100 000 people (95% CI 8·05–8·26) in 1999 to 20·48 per 100 000 (20·34–20·62) in 2000. However, both studies only reported on cause-specific mortality and did not account for changes in the prevalence of atrial fibrillation or heart failure during the same period. Previous research has reported on the poor prognosis of people with heart failure and how survival rates in the UK both in the community and after hospital discharge have remained largely static over time. Additionally, poor short-term survival among people with both heart failure and atrial fibrillation has been reported in secondary care cohorts, but not among primary care cohorts over extended follow-up.

### Added value of this study

This study provides longitudinal data and long-term follow-up on a large population of people with heart failure and/or atrial fibrillation who were diagnosed in either primary or secondary care. The data enabled us to comprehensively analyse all-cause mortality, including trends in the probability of mortality for diagnoses over time and among key subgroups defined by age, sex, and level of social deprivation. The results indicated that people with heart failure and atrial fibrillation typically have a poor outlook, with median overall survival of just over 3 years from the time both conditions had been diagnosed. Approximately 60% of people with both heart failure and atrial fibrillation were estimated to die within the first 5 years of having both diagnoses, with age-stratified overall survival similar between men and women. People with heart failure, with or without atrial fibrillation, were more likely to live in areas of social deprivation than those with neither condition, which was in turn associated with decreased overall survival. There was no improvement in the probability of all-cause mortality for people with heart failure and atrial fibrillation across successive years of diagnosis over nearly 20 years of follow-up. Additionally, in people with both conditions, an observable trend of higher mortality probability in the most deprived quintile versus the least deprived quintile did not change across follow-up. Improvements in short-term and long-term mortality probability estimates across successive years of diagnosis were observed for people diagnosed with heart failure or atrial fibrillation who did not develop the other condition, and in long-term mortality estimates among people diagnosed with both conditions before age 65 years.

### Implications of all the available evidence

Our results emphasise the increased all-cause mortality associated with comorbid atrial fibrillation and heart failure and the high prevalence of these two conditions in combination. Limited improvements in mortality occurred between 2000 and 2018 despite the development of a number of prognostically beneficial medications for people with heart failure. Health policy makers should consider strategies to improve earlier diagnosis of heart failure and the implementation of proven therapies with a view to improving survival.

## References

[1] Roth GA, Mensah GA, Johnson CO, the GBD-NHLBI-JACC Global Burden of Cardiovascular Diseases Writing Group (2020). Global burden of cardiovascular diseases and risk factors, 1990–2019: update from the GBD 2019 study. J Am Coll Cardiol.

[2] Becher PM, Lund LH, Coats AJS, Savarese G (2022). An update on global epidemiology in heart failure. Eur Heart J.

[3] Savarese G, Lund LH (2017). Global public health burden of heart failure. Card Fail Rev.

[4] Heist EK, Ruskin JN (2006). Atrial fibrillation and congestive heart failure: risk factors, mechanisms, and treatment. Prog Cardiovasc Dis.

[5] Conrad N, Judge A, Tran J (2018). Temporal trends and patterns in heart failure incidence: a population-based study of 4 million individuals. Lancet.

[6] Wang TJ, Larson MG, Levy D (2003). Temporal relations of atrial fibrillation and congestive heart failure and their joint influence on mortality: the Framingham Heart Study. Circulation.

[7] Santhanakrishnan R, Wang N, Larson MG (2016). Atrial fibrillation begets heart failure and vice versa: temporal associations and differences in preserved versus reduced ejection fraction. Circulation.

[8] Vinter N, Huang Q, Fenger-Grøn M, Frost L, Benjamin EJ, Trinquart L (2020). Trends in excess mortality associated with atrial fibrillation over 45 years (Framingham Heart Study): community based cohort study. BMJ.

[9] Taylor CJ, Ordóñez-Mena JM, Roalfe AK (2019). Trends in survival after a diagnosis of heart failure in the United Kingdom 2000–2017: population based cohort study. BMJ.

[10] Jones NR, Roalfe AK, Adoki I, Hobbs FDR, Taylor CJ (2019). Survival of patients with chronic heart failure in the community: a systematic review and meta-analysis. Eur J Heart Fail.

[11] Sartipy U, Dahlström U, Fu M, Lund LH (2017). Atrial fibrillation in heart failure with preserved, mid-range, and reduced ejection fraction. JACC Heart Fail.

[12] Zafrir B, Lund LH, Laroche C, the ESC-HFA HF Long-Term Registry Investigators (2018). Prognostic implications of atrial fibrillation in heart failure with reduced, mid-range, and preserved ejection fraction: a report from 14 964 patients in the European Society of Cardiology Heart Failure Long-Term Registry. Eur Heart J.

[13] Dries DL, Exner DV, Gersh BJ, Domanski MJ, Waclawiw MA, Stevenson LW (1998). Atrial fibrillation is associated with an increased risk for mortality and heart failure progression in patients with asymptomatic and symptomatic left ventricular systolic dysfunction: a retrospective analysis of the SOLVD trials. J Am Coll Cardiol.

[14] McManus DD, Hsu G, Sung SH, the Cardiovascular Research Network PRESERVE Study (2013). Atrial fibrillation and outcomes in heart failure with preserved versus reduced left ventricular ejection fraction. J Am Heart Assoc.

[15] Cheng M, Lu X, Huang J, Zhang J, Zhang S, Gu D (2014). The prognostic significance of atrial fibrillation in heart failure with a preserved and reduced left ventricular function: insights from a meta-analysis. Eur J Heart Fail.

[16] Weber C, Hung J, Hickling S (2021). Incidence, predictors and mortality risk of new heart failure in patients hospitalised with atrial fibrillation. Heart.

[17] Bauersachs J (2021). Heart failure drug treatment: the fantastic four. Eur Heart J.

[18] Ibrahim R, Singh VJ, Singh SJ, Hussein A, Lee JZ (2024). Atrial fibrillation mortality trends in individuals with heart failure. J Investig Med.

[19] Zuin M, Bertini M, Vitali F, Turakhia M, Boriani G (2024). Heart failure-related death in subjects with atrial fibrillation in the United States, 1999 to 2020. J Am Heart Assoc.

[20] Herrett E, Gallagher AM, Bhaskaran K (2015). Data resource profile: Clinical Practice Research Datalink (CPRD). Int J Epidemiol.

[21] Smith T, Noble M, Noble S, Wright G, McLennan D, Plunkett E (2015). The English indices of deprivation 2015: technical report.

[22] National Institute for Cardiovascular Outcomes Research (2020). National Heart Failure Audit (NHFA): 2020 summary report (2018/19 data).

[23] Fletcher RA, Rockenschaub P, Neuen BL, the CVD-COVID-UK/COVID-IMPACT Consortium (2024). Contemporary epidemiology of hospitalised heart failure with reduced versus preserved ejection fraction in England: a retrospective, cohort study of whole-population electronic health records. Lancet Public Health.

[24] Mamas MA, Caldwell JC, Chacko S, Garratt CJ, Fath-Ordoubadi F, Neyses L (2009). A meta-analysis of the prognostic significance of atrial fibrillation in chronic heart failure. Eur J Heart Fail.

[25] McMurray JJ, Packer M, Desai AS, the PARADIGM-HF Investigators and Committees (2014). Angiotensin-neprilysin inhibition versus enalapril in heart failure. N Engl J Med.

[26] McMurray JJV, Solomon SD, Inzucchi SE, the DAPA-HF Trial Committees and Investigators (2019). Dapagliflozin in patients with heart failure and reduced ejection fraction. N Engl J Med.

[27] Vaduganathan M, Docherty KF, Claggett BL (2022). SGLT-2 inhibitors in patients with heart failure: a comprehensive meta-analysis of five randomised controlled trials. Lancet.

[28] Vohra AS, Moghtaderi A, Luo Q (2024). Trends in mortality after incident hospitalization for heart failure among Medicare beneficiaries. JAMA Netw Open.

[29] Garred CH, Malmborg M, Malik ME (2024). Age-specific mortality trends in heart failure over 25 years: a retrospective Danish nationwide cohort study. Lancet Healthy Longev.

[30] McDonagh TA, Metra M, Adamo M, the ESC Scientific Document Group (2021). 2021 ESC guidelines for the diagnosis and treatment of acute and chronic heart failure. Eur Heart J.

[31] Packer M, Lam CSP, Lund LH, Redfield MM (2020). Interdependence of atrial fibrillation and heart failure with a preserved ejection fraction reflects a common underlying atrial and ventricular myopathy. Circulation.

[32] Bates BA, Akhabue E, Nahass MM (2023). Validity of International Classification of Diseases (ICD)-10 diagnosis codes for identification of acute heart failure hospitalization and heart failure with reduced versus preserved ejection fraction in a national Medicare sample. Circ Cardiovasc Qual Outcomes.

