# Supplementary material for: Trends in mortality in people with heart failure and atrial fibrillation: a population-based cohort study
Source: Lancet Healthy Longev. Author manuscript; Available in PMC 2026 Jan 30. (PMC7618696; doi:10.1016/j.lanhl.2025.100734)
Supplement: Supplementary appendix [file EMS212009-supplement-Supplementary_appendix.pdf]

THE LANCET
Healthy Longevity

Supplementary appendix

This appendix formed part of the original submission and has been peer reviewed. We post it as supplied by the authors.

Supplement to: Jones NR, Smith M, Yang Y, et al. Trends in mortality in people with heart failure and atrial fibrillation: a population-based cohort study. *Lancet Healthy Longev* 2025. https://doi.org/10.1016/j.lanhl.2025.100734

Supplemental Table 1. Mean age at time of diagnosis with heart failure, atrial fibrillation or both, comparing by gender and first and last year of study follow-up.

	Heart failure and atrial fibrillation	Atrial fibrillation	Heart failure
Whole population	79.2 (9.9)	75.9 (11.0)	77.0 (11.1)
Male	76.9 (10.0)	73.4 (10.9)	74.6 (11.2)
Female	81.7 (8.98)	78.5 (10.5)	79.6 (10.4)
Earliest year of cohort (2000)	77.7 (9.6)	74.5 (10.5)	76.6 (10.3)
Last year of cohort (2018)	78.7 (10.2)	75.5 (11.3)	76.6 (11.6)

All values are mean age in years with standard deviation in brackets

Age at diagnosis with heart failure and atrial fibrillation is based on the age diagnosed with the later condition, if diagnosed with the two conditions on separate dates

Supplemental Table 2. Proportion of patients newly diagnosed with atrial fibrillation who had a CHA2DS2VASc score ≥2 and were prescribed an oral anticoagulant at key follow-up time points

	End of follow-up after 31.12.2005		End of follow-up after 31.12.2009		End of follow-up after 31.12.2013		End of follow-up after 31.12.2017	
	Number of participants	Number prescribed OAC	Number of participants	Number prescribed OAC	Number of participants	Number prescribed OAC	Number of participants	Number prescribed OAC
**Atrial fibrillation (with or without heart failure)**	133,959	48,700 (36.3%)	104,737	41,240 (39.4%)	63,035	28,575 (45.3%)	15,880	8,784 (55.3%)
**Heart failure and atrial fibrillation**	33,144	11,951 (36.1%)	23,966	9,351 (39.0%)	13,201	5,993 (45.4%)	2,989	1,711 (57.2%)

Data are number of participants (%). OAC = oral anticoagulation

Supplemental Table 3: Cox regression model showing the relative risk of all-cause mortality based on the order in which heart failure and atrial fibrillation developed, including sub-group analysis by sex and age categories. Hazard ratio with 95% confidence interval reported.

	Number of participants at risk	Number of deaths	Overall	Sub-group analysis by sex		Sub-group analysis by age category at time of diagnosis				
				Male	Female	Age <65	Age 65-74	Age 75-84	Age 85-94	Age ≥95
**Neither Heart failure nor atrial fibrillation**	2,294,067	191,680	1 (ref)	1 (ref)		1 (ref)				
**Heart failure and atrial fibrillation diagnosed same day**	15,101	8,880	4.00 (3.92 to 4.10)	4.03 (3.90 to 4.17)	3.93 (3.82 to 4.05)	5.97 (5.46 to 6.53)	4.85 (4.59 to 5.19)	4.04 (3.90 to 4.19)	3.44 (3.31 to 3.57)	2.75 (2.48 to 3.06)
**Heart failure with prevalent atrial fibrillation**	36,150	19,434	3.36 (3.31 to 3.42)	3.28 (3.20 to 3.35)	3.41 (3.34 to 3.49)	4.53 (4.23 to 4.85)	3.90 (3.76 to 4.06)	3.40 (3.31 to 3.48)	3.10 (3.01 to 3.19)	2.38 (2.19 to 2.60
**Atrial fibrillation with prevalent heart failure**	23,219	14,113	3.79 (3.72 to 3.86)	3.88 (3.78 to 3.98)	3.66 (3.57 to 3.75)	6.35 (5.89 to 6.85)	5.13 (4.92 to 5.35)	3.83 (3.72 to 3.94)	3.02 (2.92 to 3.12)	2.73 (2.49 to 2.99)

Each model was adjusted for hypertension, diabetes, history of previous stroke, thromboembolism or vascular disease (including ischaemic heart disease, myocardial infarct, aortic plaque or peripheral arterial disease), smoking and ethnicity. The model also adjusted for age and/or sex, unless this was the focus of the sub-group analysis.

Likelihood ratio chi-square test for the interaction between sex and the order in which heart failure and atrial fibrillation were diagnosed within the fully adjusted Cox model = 700.3 ($p < 0.0001$)

Likelihood ratio chi-square test for the interaction between age category and the order in which heart failure and atrial fibrillation were diagnosed within the fully adjusted Cox model = 54322.6 ($p < 0.0001$)

Supplemental Table 4. Median survival times for people with an incident diagnosis of heart failure, atrial fibrillation or both, by age group, gender and IMD quintiles as a measure of deprivation status. All survival times are reported in years.

Group	Subgroup	**Heart failure without atrial fibrillation at the time of diagnosis**			**Atrial fibrillation without heart failure at the time of diagnosis**			**Heart failure and atrial fibrillation**		
		Overall	Age <75	Age ≥75	Overall	Age <75	Age ≥75	Overall	Age <75	Age ≥75
Overall		4.08 (4.01 to 4.14)			6.55 (6.46 to 6.65)			3.15 (3.08 to 3.21)		
Sex	Male	4.52 (4.42 to 4.61)	9.07 (8.82 to 9.40)	2.44 (2.36 to 2.51)	7.36 (7.23 to 7.49)	13.6 (13.4 to 14.1)	3.68 (3.58 to 3.77)	3.52 (3.42 to 3.63)	7.21 (6.93 to 7.57)	2.08 (1.99 to 2.17)
	Female	3.65 (3.56 to 3.73)	8.53 (8.17 to 8.91)	2.61 (2.54 to 2.67)	5.86 (5.73 to 5.97)	13.6 (13.2 to 14.1)	3.81 (3.73 to 3.91)	2.80 (2.71 to 2.90)	6.56 (6.19 to 7.18)	2.13 (2.05 to 2.23)
Age at diagnosis	< 65	13.6 (13.1 to 14.8)			N/A			11.7 (10.9 to 13.3)		
	65-74	7.01 (6.81 to 7.19)			10.6 (10.4 to 10.8)			5.66 (5.44 to 5.94)		
	≥75	2.53 (2.48 to 2.58)			3.75 (3.69 to 3.81)			2.11 (2.05 to 2.17)		
Indices of deprivation quintile	1st (least deprived)	4.34 (4.20 to 4.49)	10.6 (9.99 to 11.2)	2.80 (2.69 to 2.94)	8.13 (7.89 to 8.34)	16.1 (15.6 to 16.6)	4.35 (4.22 to 4.47)	3.46 (3.31 to 3.59)	8.68 (7.81 to 9.67)	2.34 (2.20 to 2.46)
	2nd	4.18 (4.03 to 4.33)	9.71 (9.34 to 10.2)	2.66 (2.57 to 2.77)	7.07 (6.92 to 7.26)	14.9 (14.3 to 15.4)	4.00 (3.89 to 4.15)	3.25 (3.12 to 3.40)	7.86 (7.18 to 8.73)	2.17 (2.07 to 2.31)
	3rd	4.09 (3.94 to 4.22)	9.51 (8.99 to 10.1)	2.46 (2.34 to 2.57)	6.62 (6.44 to 6.84)	13.9 (13.2 to 15.1)	3.73 (3.60 to 3.90)	3.30 (3.14 to 3.45)	7.69 (6.87 to 8.26)	2.16 (2.02 to 2.31)
	4th	3.89 (3.73 to 4.03)	8.25 (7.85 to 8.61)	2.38 (2.28 to 2.48)	5.56 (5.39 to 5.72)	11.7 (11.2 to 12.2)	3.33 (3.17 to 3.46)	2.97 (2.84 to 3.12)	6.26 (5.75 to 6.77)	1.89 (1.77 to 2.08)
	5th (most deprived)	3.85 (3.69 to 3.99)	7.00 (6.67 to 7.35)	2.29 (2.18 to 2.40)	4.74 (4.55 to 4.95)	9.25 (8.78 to 9.74)	2.89 (2.73 to 3.00)	2.67 (2.51 to 2.81)	5.07 (4.50 to 5.48)	1.83 (1.65 to 1.98)

Median survival times are reported in years presented with 95% confidence intervals in brackets.

*There were too few deaths among the population aged under 65 with AF alone to provide reliable estimates of survival.

Heart failure and atrial fibrillation were also included as time-varying covariates to allow individuals to move between exposure groups across follow-up. People initially diagnosed with heart failure or atrial fibrillation alone but who subsequently developed the other condition were included in the 'heart failure only' and 'atrial fibrillation only' groups respectively.

Supplemental Table 5. Median difference in the cumulative probability of mortality between people diagnosed with heart failure and/or atrial fibrillation in the year 2000 compared to people diagnosed with the same condition in the latest year of study follow-up, stratified by age and sex

Group	Subgroup	3 month	1 year	2 years	5 years	10 years
Heart failure and atrial fibrillation	Overall	-1.51 (-4.36 to 1.34)	-1.11 (-4.28 to 2.06)	-1.72 (-5.08 to 1.63)	-0.14 (-3.53 to 3.26)	-0.93 (-4.01 to 2.14)
	Women	-0.39 (-4.68 to 3.90)	0.37 (-4.30 to 5.04)	-0.61 (-5.44 to 4.22)	5.24 (0.34 to 10.1)	1.45 (-3.03 to 5.93)
	Men	-2.23 (-6.04 to 1.58)	-2.04 (-6.37 to 2.28)	-2.52 (-7.18 to 2.14)	-4.89 (-9.56 to -0.22)	-3.01 (-7.30 to 1.28)
	Age <65	0.00 (-5.92 to 5.92)	-4.84 (-12.0 to 2.30)	-7.87 (-16.2 to 0.47)	-11.6 (-22.0 to -1.17)	-14.5 (-25.2 to -3.77)
	Age 65-74	0.30 (-4.76 to 5.37)	2.65 (-3.40 to 8.71)	0.36 (-6.53 to 7.15)	-2.69 (-9.74 to 4.36)	-6.13 (-13.8 to 1.53)
	Age ≥75	-2.32 (-6.02 to 1.39)	-1.87 (-5.88 to 2.14)	-2.25 (-6.38 to 1.89)	0.46 (-3.52 to 4.44)	1.38 (-1.87 to 4.63)
Atrial fibrillation without heart failure at diagnosis ("AF only")	Overall	-0.54 (-2.18 to 1.10)	-2.35 (-4.20 to -0.51)	-1.61 (-3.66 to 0.44)	-2.03 (-4.25 to 0.19)	-0.80 (-3.09 to 1.49)
	Women	0.91 (-1.64 to 3.46)	-1.74 (-4.44 to 0.96)	0.63 (-2.33 to 3.59)	-0.28 (-3.52 to 2.96)	-1.18 (-3.52 to 2.96)
	Men	-1.02 (-3.06 to 1.02)	-1.79 (-4.24 to 0.67)	-2.47 (-5.19 to 0.25)	-2.60 (-5.58 to 0.38)	-0.71 (-3.94 to 2.52)
	Age <65	0.35 (-2.13 to 2.83)	-1.96 (-3.88 to 0.97)	-2.25 (-5.68 to 1.19)	-4.37 (-8.24 to -0.50)	-1.01 (-5.80 to 3.78)
	Age 65-74	-0.15 (-2.67 to 2.37)	0.57 (-2.51 to 3.64	-0.31 (-3.77 to 3.15)	-2.33 (-6.17 to 1.51)	-3.02 (-7.56 to 1.51)
	Age ≥75	-0.48 (-2.92 to 1.97)	-2.63 (-5.32 to 0.06)	-1.24 (-4.12 to 1.64)	-1.30 (-4.35 to 1.76)	-0.51 (-3.39 to 2.37)
Heart failure without atrial fibrillation at diagnosis ("HF only")	Overall	-1.58 (-3.58 to 0.42)	-1.63 (-3.81 to 0.55)	-2.79 (-5.14 to -0.45)	-2.39 (-4.81 to 0.03)	-3.76 (-6.08 to -1.44)
	Women	-1.58 (-4.44 to 1.28)	0.08 (-3.08 to 3.24)	-1.12 (-4.50 to 2.26)	-0.28 (-3.77 to 3.22)	-0.29 (-3.66 to 3.08)
	Men	-1.52 (-4.32 to 1.27)	-3.19 (-6.22 to -0.15)	-4.26 (-7.53 to -0.99)	-4.27 (-7.66 to -0.88)	-6.56 (-9.79 to -3.33)
	Age <65	-0.64 (-4.51 to 3.23)	-4.81 (-8.91 to -0.71)	-8.70 (-13.1 to -4.23)	-6.74 (-12.4 to -1.11)	-11.7 (-18.0 to -5.34)
	Age 65-74	-1.88 (-5.48 to 1.72)	-0.54 (-4.75 to 3.67)	-1.54 (-6.23 to 3.15)	-8.67 (-13.5 to -3.83)	-10.8 (-15.9 to -5.69)
	Age ≥75	-1.79 (-4.60 to 1.02)	-1.01 (-4.04 to 2.02)	-0.99 (-4.19 to 2.21)	0.89 (-2.17 to 3.94)	1.09 (-1.33 to 3.51)

Heart failure and atrial fibrillation were also included as time-varying covariates to allow individuals to move between exposure groups across follow-up. People initially diagnosed with heart failure or atrial fibrillation alone but who subsequently developed the other condition were included in the 'heart failure only' and 'atrial fibrillation only' groups respectively.

Supplemental Figure 1. Flow chart summarising the cohort and the number of patients diagnosed with heart failure and/or atrial fibrillation during the study period

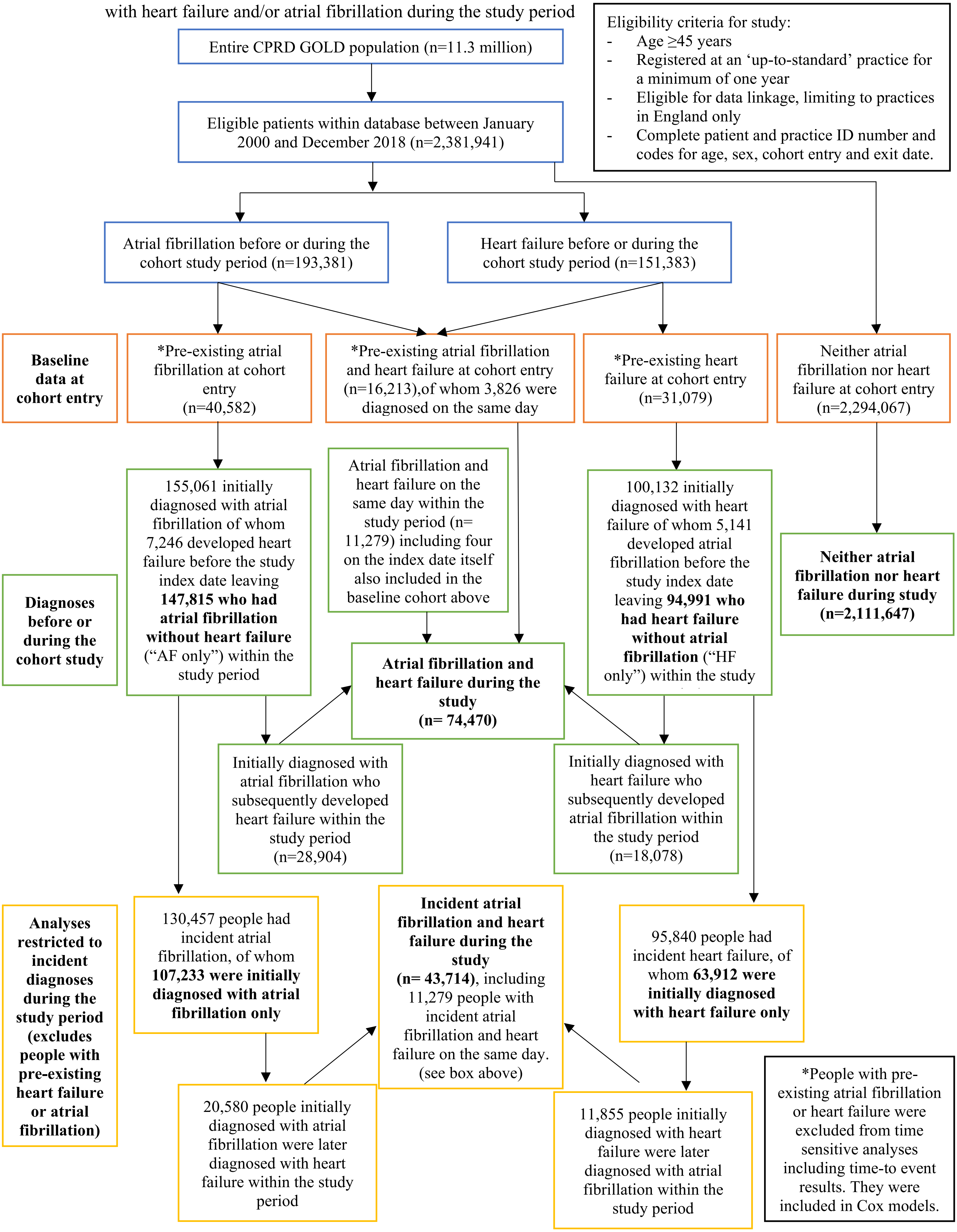

Supplemental Figure 2. Trends in cumulative probability of all-cause mortality at 3-month, 1-, 2-, 5- and 10-year follow-up for people with heart failure, based on year of diagnosis between 2000 and 2018. These patients could not have atrial fibrillation at the time of the heart failure diagnosis but may have developed it subsequently. Bars demonstrate 95% confidence intervals

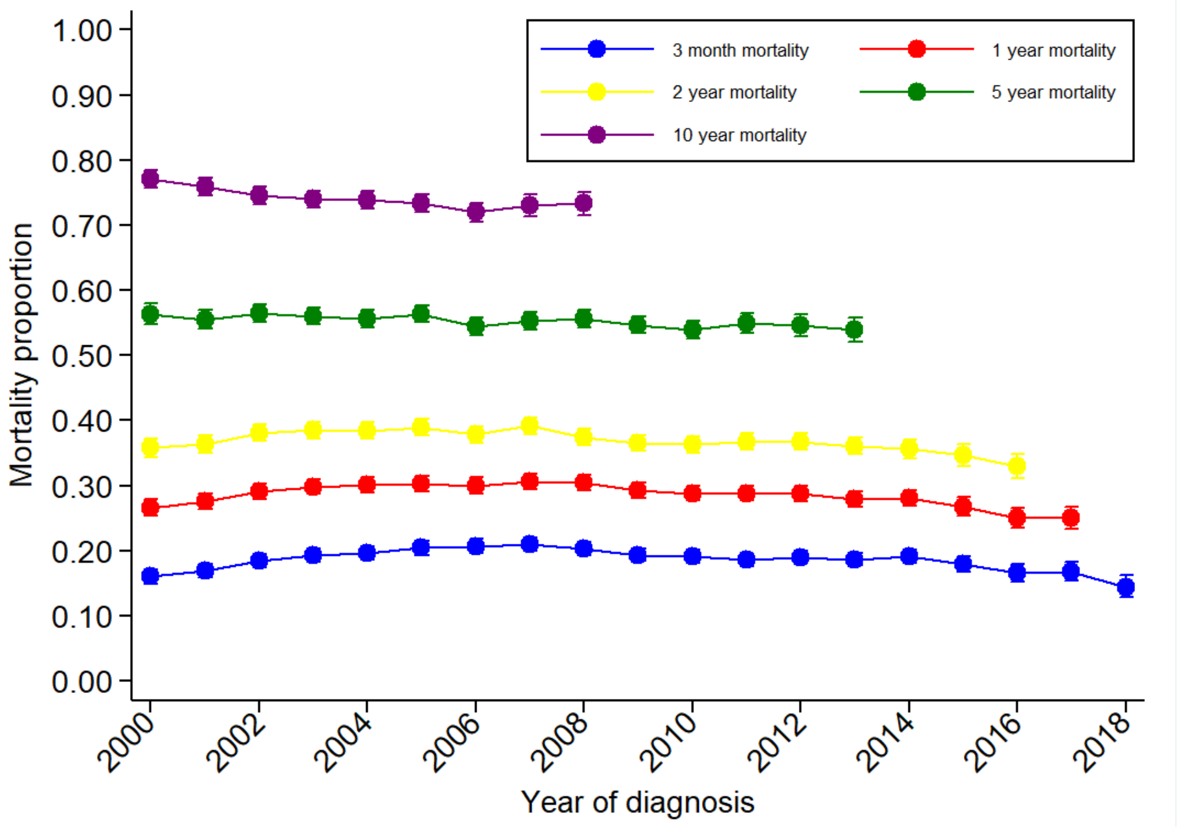

Supplemental Figure 3. Trends in cumulative probability of all-cause mortality at 3-month, 1-, 2-, 5- and 10-year follow-up for people with atrial fibrillation, based on year of diagnosis between 2000 and 2018. These patients could not have heart failure at the time of the atrial fibrillation diagnosis but may have developed it subsequently. Bars demonstrate 95% confidence intervals

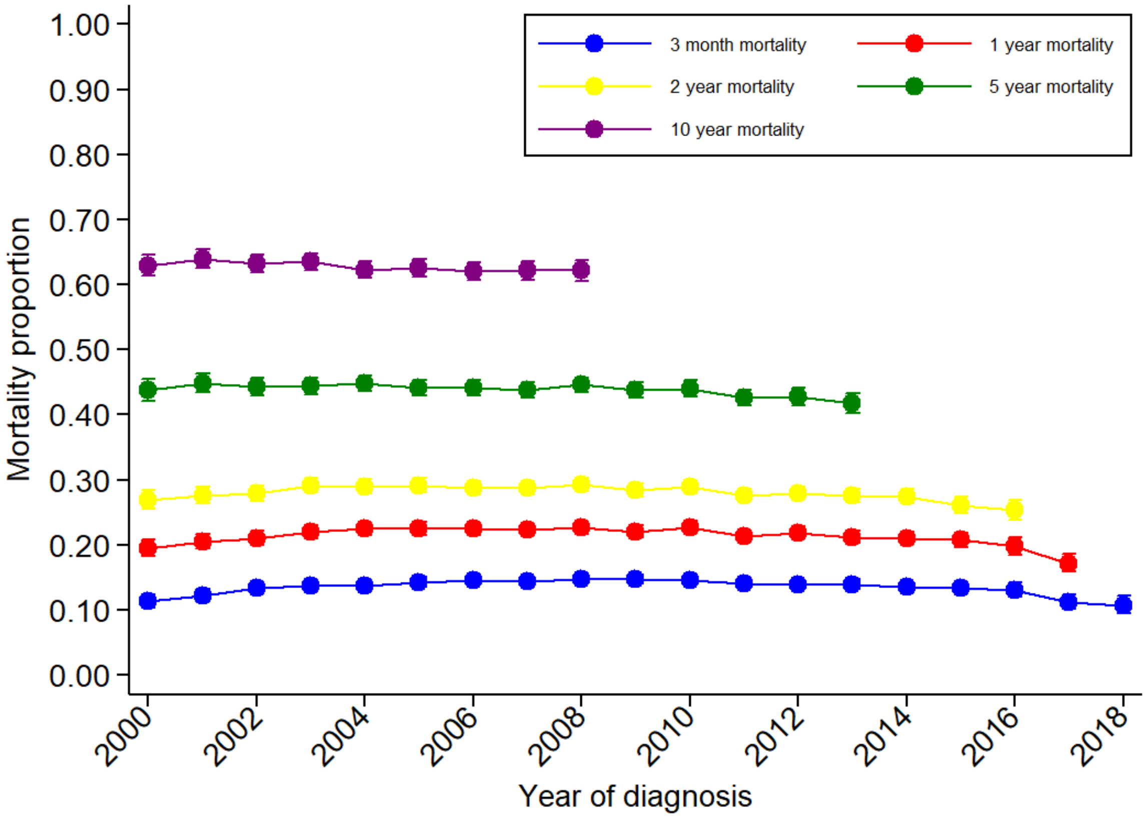

Supplemental Figure 4. Trends in cumulative probability of all-cause mortality at 3-month, 1-, 2-, 5- and 10-year follow-up for people with heart failure, based on year of diagnosis between 2000 and 2018. Patients with atrial fibrillation at any point during follow-up were excluded from this analysis. Bars demonstrate 95% confidence intervals

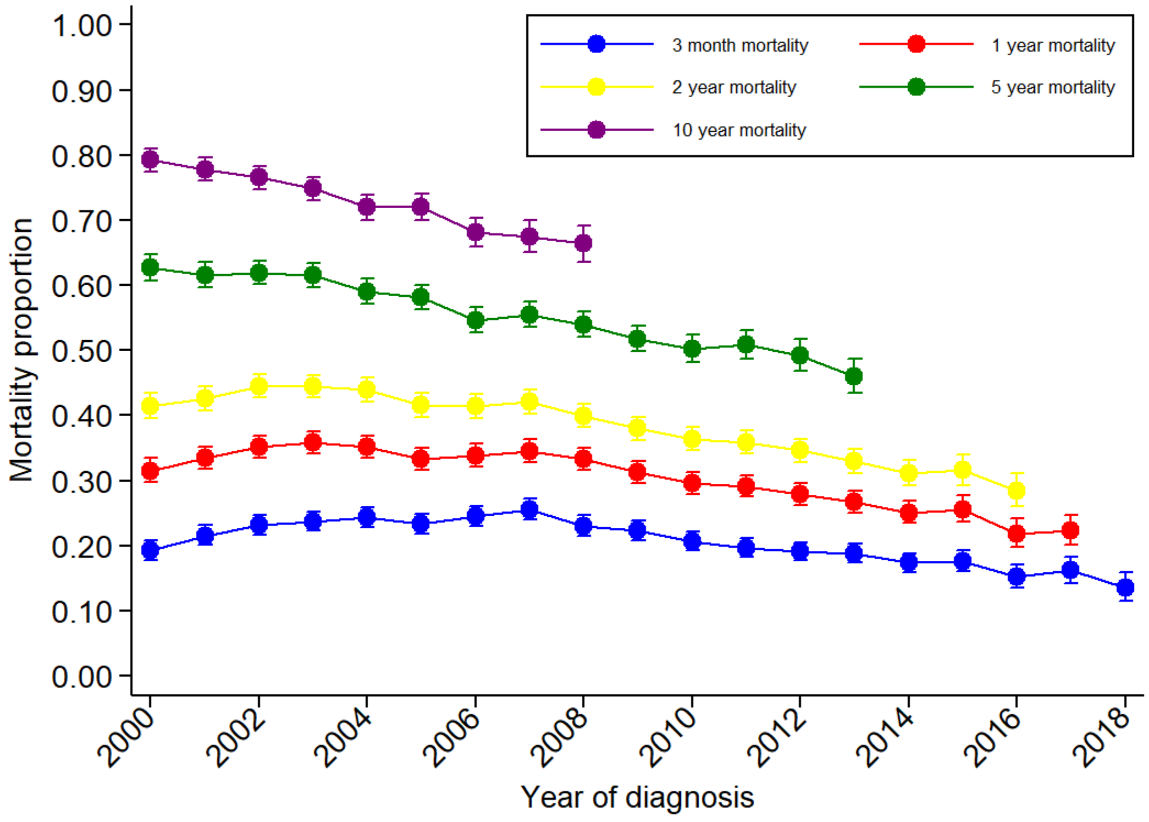

Supplemental Figure 5. Trends in cumulative probability of all-cause mortality at 3-month, 1-, 2-, 5- and 10-year follow-up for people with atrial fibrillation, based on year of diagnosis between 2000 and 2018. Patients with heart failure at any point during follow-up were excluded from this analysis. Bars demonstrate 95% confidence intervals

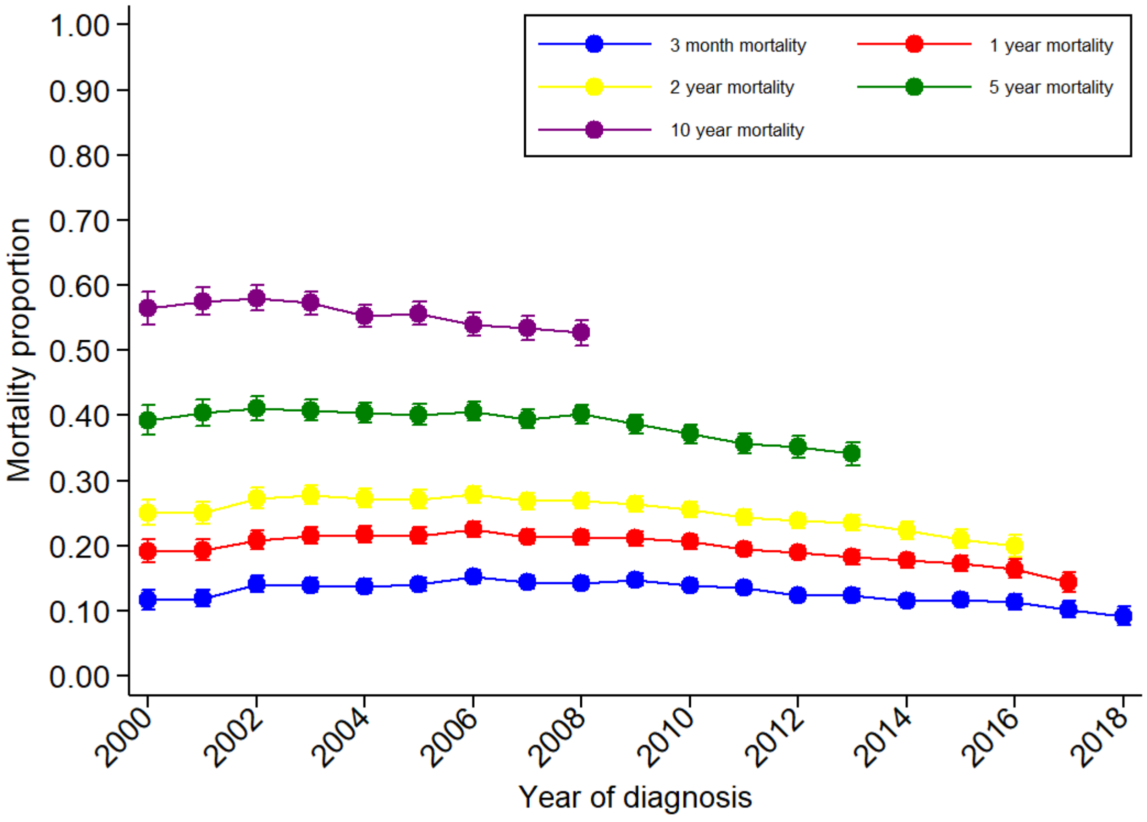

Appendix: Codes used to identify exposure variables for primary care cohort studies

**Atrial fibrillation (CPRD)**		
Description	medcode	READcode
Paroxysmal atrial fibrillation	1268	G573200
Permanent atrial fibrillation	96277	G573400
Non-rheumatic atrial fibrillation	35127	G573300
Atrial fibrillation	1664	G573000
Persistent atrial fibrillation	96076	G573500
Atrial fibrillation and flutter	2212	G573.00
Atrial fibrillation and flutter NOS	23437	G573z00
ECG: atrial fibrillation	3757	3272
Description	ICD10	
Atrial fibrillation and flutter	I48	
Paroxysmal atrial fibrillation	I48.0	
Persistent atrial fibrillation	I48.1	
Chronic atrial fibrillation	I48.2	
Typical atrial flutter	I48.3	
Atypical atrial flutter	I48.4	
"Atrial fibrillation and atrial flutter, unspecified"	I48.9	
**Heart failure (CPRD)**		
readterm	medcode	readcode
H/O: heart failure	15058	14A6.00
H/O: Heart failure in last year	46912	14AM.00
Heart failure confirmed	9913	1O1..00
On optimal heart failure therapy	111428	2JZ..00
New York Heart Assoc classification heart failure symptoms	46672	388D.00
Heart failure self-management plan agreed	106198	661M500
Heart failure 6 month review	83502	662p.00
Congestive heart failure monitoring	12366	662T.00
Heart failure annual review	30779	662W.00
Education about deteriorating heart failure	105002	679W100
Preferred place of care for next exacerbation heart failure	105542	8CeC.00
Heart failure care plan discussed with patient	32945	8CL3.00
Has heart failure management plan	103732	8CMK.00
Heart failure clinical pathway	106008	8CMW800
Admit heart failure emergency	32898	8H2S.00
Heart failure follow-up	17851	8HBE.00
Discharge from practice nurse heart failure clinic	91288	8Hg8.00
Discharge from heart failure nurse service	102585	8HgD.00
Referral to heart failure nurse	26115	8HHb.00
Referral to heart failure exercise programme	70619	8HHz.00
Referred to heart failure education group	71235	8Hk0.00
Referral to heart failure clinic	48897	8HTL.00
Referral to rapid access heart failure clinic	106680	8HTL000
Heart failure rehabilitation programme not available	110101	8I98.00
Referral to heart failure exercise programme not indicated	106836	8IB8.00
Referral to heart failure education group declined	107981	8IE0.00
Referral to heart failure exercise programme declined	106894	8IE1.00
Seen in heart failure clinic	12627	9N0k.00
Seen by community heart failure nurse	19002	9N2p.00
Did not attend practice nurse heart failure clinic	95021	9N4s.00

Did not attend heart failure clinic	83481	9N4w.00
Referred by heart failure nurse specialist	69062	9N6T.00
Heart failure monitoring administration	32911	9Or..00
Heart failure review completed	19380	9Or0.00
Heart failure monitoring telephone invite	90193	9Or1.00
Heart failure monitoring verbal invite	90192	9Or2.00
Heart failure monitoring first letter	72965	9Or3.00
Heart failure monitoring second letter	72386	9Or4.00
Heart failure monitoring third letter	89650	9Or5.00
Hypertensive heart & renal dis with (congestive) heart failure	21837	G232.00
Heart failure	2062	G58..00
Cardiac failure	1223	G58..11
Congestive heart failure	398	G580.00
Congestive cardiac failure	2906	G580.11
Right heart failure	10079	G580.12
Right ventricular failure	10154	G580.13
Biventricular failure	9524	G580.14
Chronic congestive heart failure	32671	G580100
Decompensated cardiac failure	27884	G580200
Compensated cardiac failure	11424	G580300
Congestive heart failure due to valvular disease	94870	G580400
Left ventricular failure	884	G581.00
Heart failure with normal ejection fraction	101138	G583.00
HFNEF - heart failure with normal ejection fraction	101137	G583.11
Heart failure with preserved ejection fraction	106897	G583.12
Heart failure NOS	4024	G58z.00
Cardiac failure NOS	17278	G58z.12
Post cardiac operation heart failure NOS	96799	G5y4z00
Heart failure as a complication of care	66306	SP11111
New York Heart Assoc classification heart failure symptoms	26242	ZRad.00
Cardiac failure therapy	24503	8B29.00
Excepted heart failure quality indicators: Patient unsuitable	30749	9hH0.00
Exception reporting: heart failure quality indicators	90935	9hH..00
Heart failure resolved	100784	2126400
Heart failure information given to patient	60099	67D4.00
Excepted heart failure quality indicators: Informed dissent	64062	9hH1.00
Suspected heart failure	21235	1J60.00
Acute congestive heart failure	23707	G580000
Acute heart failure	27964	G582.00
Cardiac failure following abortive pregnancy	112120	L09y200
Right ventricular failure	104275	G584.00
Impaired left ventricular function	5942	G581.13
Acute left ventricular failure	5255	G581000
New York Heart Association classification - class II	13189	662g.00
New York Heart Association classification - class I	18853	662f.00
New York Heart Association classification - class IV	51214	662i.00
New York Heart Association classification - class III	19066	662h.00

Description	ICD10
Heart failure	I50
Congestive heart failure	I50.0
Left ventricular failure	I50.1
"Heart failure, unspecified"	I50.9

Hypertensive heart disease with (congestive) heart failure	I11.0	
Hypertensive heart and renal disease with (congestive) heart failure	I13.0	
Hypertensive heart and renal disease with both (congestive) heart failure and renal failure	I13.2	
